# *Deverra tortuosa* (Desf.) DC from Saudi Arabia as a new source of marmin and furanocoumarins derivatives with α-glucosidase, antibacterial and cytotoxic activities

**DOI:** 10.1016/j.heliyon.2021.e06656

**Published:** 2021-04-05

**Authors:** Mohamed Habib Oueslati, Arbi Guetat, Jalloul Bouajila, A. Khuzaim Alzahrani, Jamith Basha

**Affiliations:** a Northern Border University, College of Sciences, Department of Chemistry, Saudi Arabia; b University of Carthage, Faculty of Science Bizerte, Laboratory of Hetero-Organic Compounds and Nanostructured Materials, Zarzouna, Tunisia; c Northern Border University, College of Sciences, Department of Biological Sciences, Saudi Arabia; d University of Carthage, National Institute of Applied Science and Technology, Department of Biology, Laboratory of Plant Biotechnology, B.P. 676, 1080 Tunis Cedex, Tunisia; e Laboratoire de Génie Chimique, Université de Toulouse, CNRS, INPT, UPS, Toulouse, France; f Northern Border University, College of Applied Medical Sciences, Arar, Saudi Arabia

**Keywords:** *Deverra tortuosa*, Marmin, Furanocoumarins, Cytotoxic activity, α-Glucosidase inhibition, Antibacterial activities

## Abstract

*Deverra tortuosa* (Desf.) DC (Syn. *Pituranthos tortusus* (Desf.) Benth. & Hook.f.) is a species belonging to the Apiaceae family that is common in the Northern Region of Saudi Arabia. The plant is well known in traditional medicine along the Arabian ecoregion. In the framework of the present study, the crude extract of n-hexane and ethyl acetate of the seeds were fractionated to purify major bioactive secondary metabolites. Five compounds were identified for the first time from the seeds of *D. tortuosa*: Marmin **1**, Pituranthoside **2**, Isoimperatorin **3**, Bergapten **4** and Isopimpinellin **5**. Their structures were elucidated using 1D and 2D NMR, (ESI)-MS and IR spectroscopic analyses. The cytotoxic, α-glucosidase and antibacterial activities of the pure phytochemicals were also evaluated.

## 1 Introduction

**Figure 1:**
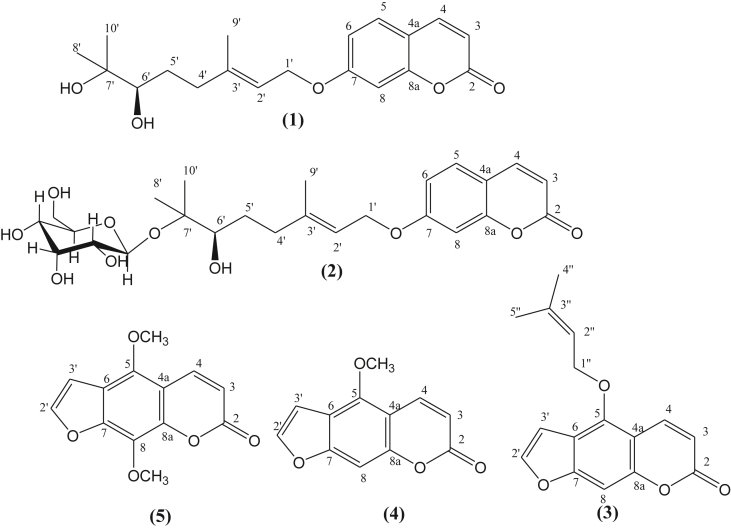
Coumarins (1: Marmin; 2: Pituranthoside) and furanocoumarins (3: Isoimperatorin; 4: Bergapten; 5: Isopimpinellin) isolated from seeds of *D. tortuosa.*

*Deverra tortuosa* (Syn. *Pituranthos tortuosus*) is distributed over a large area in the Northern Region of Saudi Arabia (Guetat et al., 2019). The plant is widely widespread in Northern Africa, and Arabia (Boulos, 2002) and is well known in traditional medicine along the Arabian ecoregion (Assy et al., 2019). Furthermore, many authors have reported the plant as a source for many purposes. Among others, *D*. *tortuosa* is used for medicinal, aromatic, edible food and fuel wood (Bidak et al., 2015). In many countries in the Arabian ecoregion, tender shoots and aerial parts of the plant are used for the treatment of hypertension and as diuretics, carminatives and analgesics, among other uses (Assy et al., 2019; Hammoudi et al., 2018; Mahran et al., 1989). Phytochemically, previous studies on this species have focused mainly on organic extracts of the aerial parts, roots and seeds (Abdelwahed et al., 2008; Guetat et al., 2019). However, the essential oils of different parts (stem, flowers, roots and seeds) of the species have been well investigated, as well as their biological activities (Guesmi et al., 2017; Aloui and Zouari, 2019; Abdelwahed et al., 2006; Guetat et al., 2019; Krifa et al., 2015, 2016; Mighri et al., 2015; Abdelghany and Hafez., 1995). Previous phytochemical studies of *D. tortuosa* were limited to the biological activities of extracts and essential oils. The present study, focused on the phytochemicals extracted from the seeds of the species. Pure compounds of organic extracts of powdered seeds were purified and characterized (Figure 1). In the second part of the present investigation, the cytotoxic, α-glucosidase, antibacterial and cytotoxic activities of the identified pure compounds were tested.

## 2 Materials and methods

### 2.1 General experimental procedures

The mom-dimensional (500 MHz) and two-dimensional nuclear magnetic resonance (NMR) spectra of the compounds were recorded in chloroform-d and methanol-d_4_ using a JEOL JNM ECX 500 NMR spectrometer with tetramethylsilane (TMS) as a resonance internal standard. The coupling constants are expressed in Hertz. The chemical shifts are expressed in δ parts per million (ppm). The IR spectra were established using a Thermo Scientific Nicolet iS5 Infrared Spectrometer in KBr. Electrospray (ESI)-MS spectra were obtained by using an ultra-performance liquid chromatography (UPLC) Xevo G2 Q time-of-flight (TOF) system (Waters).

### 2.2 Plant material

In September 2017, *Deverra tortuosa* seeds were collected during the fructification period from the wild in Wadi Abalkour (30°.83′02″ N, 41°.24′53‴ E) situated 50 km East of Arar city (Northern Border Region of Saudi Arabia). The plant specimen was identified by Dr. A. Guetat, and a voucher specimen (Dto 911) was deposited in herbarium of the College of Science Northern Border University (HCSNBU).

### 2.3 Extraction and isolation

**Figure 2:**
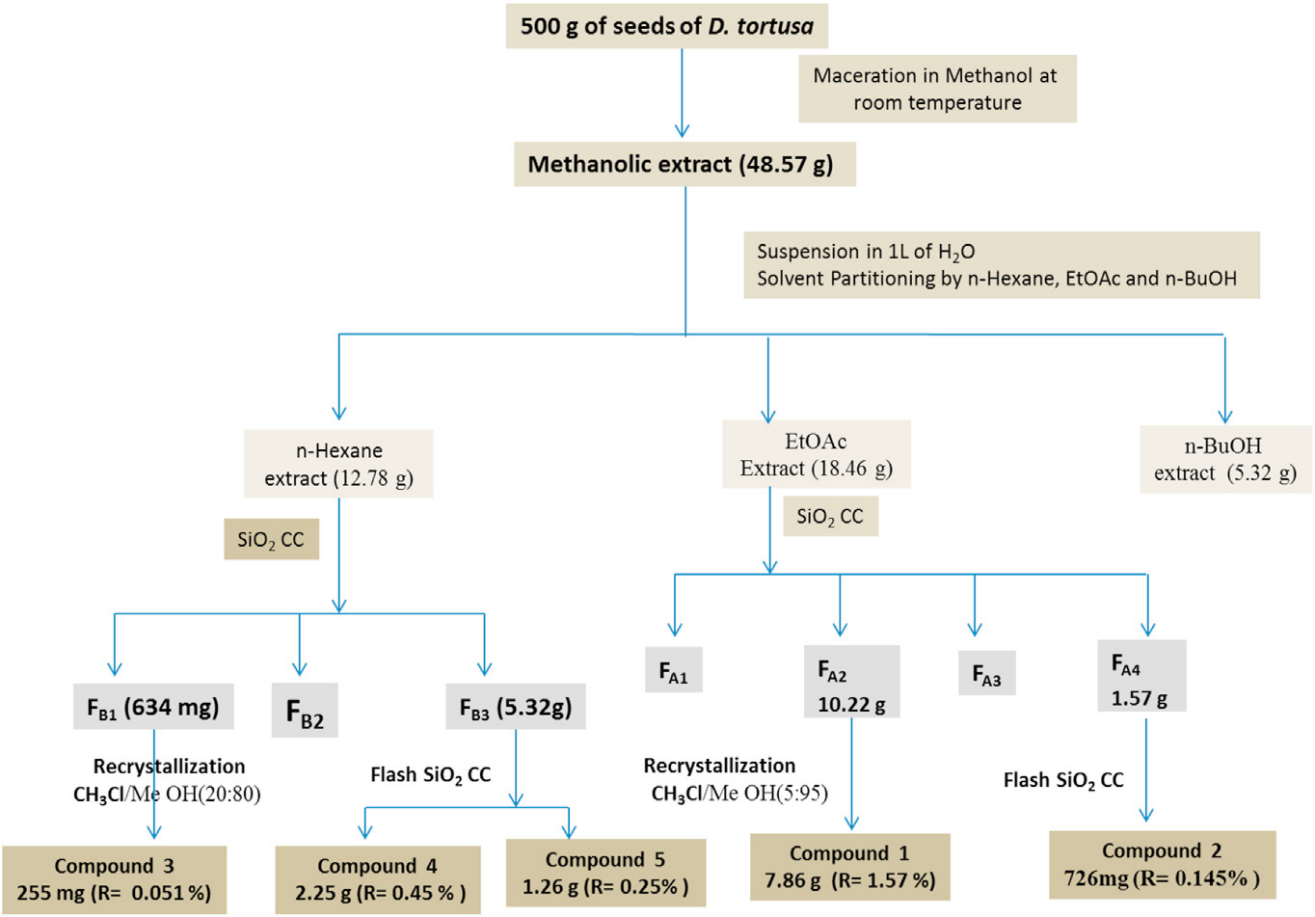
The isolation procedure of pure compounds from seeds of *D*. *tortuosa*.

Prior to maceration, the collected seeds of *D. tortuosa* were dried in the shade for 4 weeks. Seed powder (500 g) was macerated for 3 × 48 h with methanol. The obtained extract was filtered and then evaporated by rotary evaporator to yield a pale brown crude extract (48.57 g). The extract was dissolved in H_2_O (1 L) and then successively partitioned with n-hexane (3 × 1 L), ethyl acetate (3 × 1 L), and n-BuOH (3 × 500 mL). The ethyl acetate extract (18.46 g) was separated over a Si gel column eluted with n-hexane-ethyl acetate-methanol mixtures of increasing polarity n-hexane/ethyl acetate (80:20–100% ethyl acetate) and ethyl acetate /methanol (95:5–100% MeOH) to obtain four fractions, F_A1_–F_A4_. The major compound (**1**, m = 7.86 g) was obtained by recrystallization of fraction F_A2_ (10.22 g) in chloroform/methanol (5:95). Purification by flash column chromatography of fraction F_A4_ (523 mg) using CH_3_Cl/MeOH (80:20) yielded compound **2** (726 mg). The n-hexane extract (12.78 g) was purified by over an Si gel column eluted with n-hexane-chloroform of increasing polarity (90:10–100% CHCl_3_) to yield three fractions (F_B1_–_B3_). Purification of fraction F_B1_ by recrystallization allowed compound **3** (255 mg). Compounds **4** (2.25 g) and **5** (1.26 g) were obtained from fraction F_B3_ by silica gel flash column chromatography using n-hexane/CH_3_Cl as the eluent (70:30) (Figure 2).

### 2.4 α-Glucosidase inhibitory activity

The protocol for α-glucosidase inhibitory test (compounds **1**–**5**) was used as, previously, described by Tao et al. (2013). The substrate P-nitrophenyl-α-D-glucopyranoside (PNPG) was used for this experiment. Dimethyl sulfoxide (DMSO) was used to prepare different concentrations of the compounds (10–1000 μM). In brief, 20 μL of the tested compounds were mixed with 20 μL of the enzyme solution [0.1 U/mL α-glucosidase in phosphate buffer (pH 6.8)]. Afterwards, 40 μL of PNPG solution (0.375 mM) prepared in phosphate buffer (pH = 6.8) was added to the mixture and then incubated (37 °C for 30 min). 80 μL of Na_2_CO_3_ (0.2 M) prepared in phosphate buffer was added to the mixture. The absorbance was measured at 405 nm, and the measurement was replicated 3 times. The percentage of inhibition (%) reflects the α-glucosidase inhibitory activity and was calculated by formula (I):

(I) α-glucosidase inhibition activity (%) = [(A_control_ – A_sample_)/A_control_] × 100

where A_sample_ is the absorbance (405 nm) in the presence of the isolated compound and A_control_ is the absorbance (405 nm) of the control (A control consists of 20 μL potassium phosphate buffer instead of α-glucosidase solution).

### 2.5 Antibacterial activities

#### 2.5.1 Microorganisms

ATCC strains (American Type Culture Collection) were purchased from BD (Becton, Dickinson and Company). These strains were primarily activated in brain heart infusion broth and then grown on nonselective media nutrient agar (NA). A total of 7 bacterial strains (*Escherichia*. *coli*: ATCC 25922, *Klebsiella pneumonia*: ATCC 700603, *Proteus mirabilis*: ATCC 14153, *Salmonella typhimurium*: ATCC 14028, *Pseudomonas aeruginosa*: ATCC 254992, *Staphylococcus aureus*: ATCC 254996 and *Enterococcus faecalis*: ATCC 29212) were tested for antibacterial activity.

#### 2.5.2 Screening for antibacterial activity

The 0.5 Macfarland standards were used to prepare the bacterial inoculum from log phase bacterial growth. Muller-Hinton agar (MHA) media plates were prepared aseptically in a safety cabinet; the medium was used to evaluate antibacterial activity. Sterile cork borer was used to make five wells (6 mm). MHA medium was swabbed with fresh culture (24 h ATCC bacterial strains). 50 μL of the tested compounds (0.5 mg/mL) prepared in DMSO were dispensed in the wells. As a positive control, 15 μg of tetracycline was used and the negative control consists of 50 μL DMSO. The test was conducted with 3 replications. The plates were incubated according to Clinical and Laboratory Standards Institute guidelines (CLSI 2017) and the experiments were carried out aerobically at 35–37 °C for 18–20 h. The diameter of the inhibition zone around the wells was measured using a ruler, and the mean and standard deviation were calculated for three readings.

### 2.6 Cytotoxic activity assay

#### 2.6.1 Tested cell lines

In the present study, two cancer cell lines were tested: HCT116 (colorectal carcinoma) and MCF-7 (breast cancer) (American Type Culture Collection, USA). Fetal calf serum (FCS: 10 %) and L-glutamine (2 mM) were used to make complete growth medium to conserve the cell lines (RPMI: Roswell Park Memorial Institute, France). Cell lines were incubated at 37 °C under atmosphere conditions of CO_2_ (5%). All tests were performed when the cells reached 80% confluence, and a trypan blue exclusion assay was used to test the viability of the cell lines (Louis and Siegel, 2011).

#### 2.6.2 Measuring cell viability (MTT assay)

The cytotoxicity of the isolated compounds against HCT116 (colorectal carcinoma) and MCF-7 (breast cancer) cells was evaluated spectrophotometrically through an MTT assay. The assay was performed in 96-well microtiter plates (Bekir et al., 2013). The concentrations of HCT-116 and MCF-7 cells were fixed at 10 × 10^3^ and 12 × 10^3^ cells/well, respectively. The adherent cells were incubated overnight (37 °C) in a 5% enriched CO_2_ atmosphere. For the cells in the exponential growth phase, further incubation (37 °C) was maintained for 72 h, and the concentration of the tested compounds was 100 μM (DMSO does not exceed 1%). After removing the medium, incubated cells (37 °C) were mixed with 50 *μ*L of MTT solution (3 mg/mL in phosphate-buffered saline, PBS) for 20–40 min. The dissolution of the cell mitochondria and the precipitation of violet formazan crystals proceeded by adding 80 *μ*L of 100% DMSO. The measurement of the obtained reaction was taken spectrophotometrically at 540 nm. As a positive control, Doxorubicin, a conventional anticancer drug, was used. Cell viability was measured using formula II:

II % Inhibition = 100∗[(Abs _control_ – Abs _blank control_)/(Abs _sample_- Abs _blank sample)_]

where Abs _control_ is the absorbance measured of the total cell activity as described without any inhibition; Abs _blank control_ is the absorbance of MTT substrate; and Abs _sample_ is the absorbance of each inhibitor compound sample.

## 3 Results and discussion

### 3.1 Structural elucidation of compounds 1–5

The methanolic extract of seeds of *D. tortuosa* was suspended in H_2_O and successively partitioned with n-hexane, yielding the n-hexane extract and the EtOAc extract. The successive chromatographic purification of the last two extracts over a silica gel column afforded five compounds (1–5) for the first time in *D. tortuosa*: two monoterpenoid coumarins in ethyl acetate extract, Marmin (**1**), Pituranthoside (**2**), and three furanocoumarins, Isoimperatorin (**3**) Bergapten (**4**) and Isopimpinellin (**5**), in n-hexane extract. The structures of isolated compounds were established by a combination of methods, such as 1D and 2D NMR, IR and mass spectrometry.

#### 3.1.1 Marmin (1)

**Figure 3:**
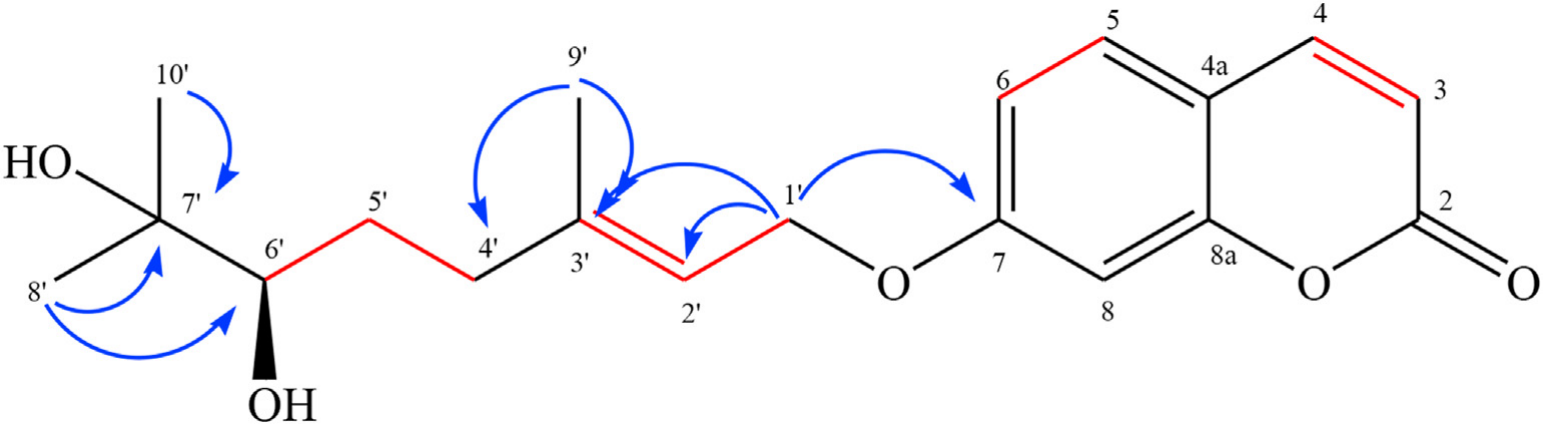
Relevant COSY () and HMBC (H C) correlations in Marmin (1).

**Figure 4:**
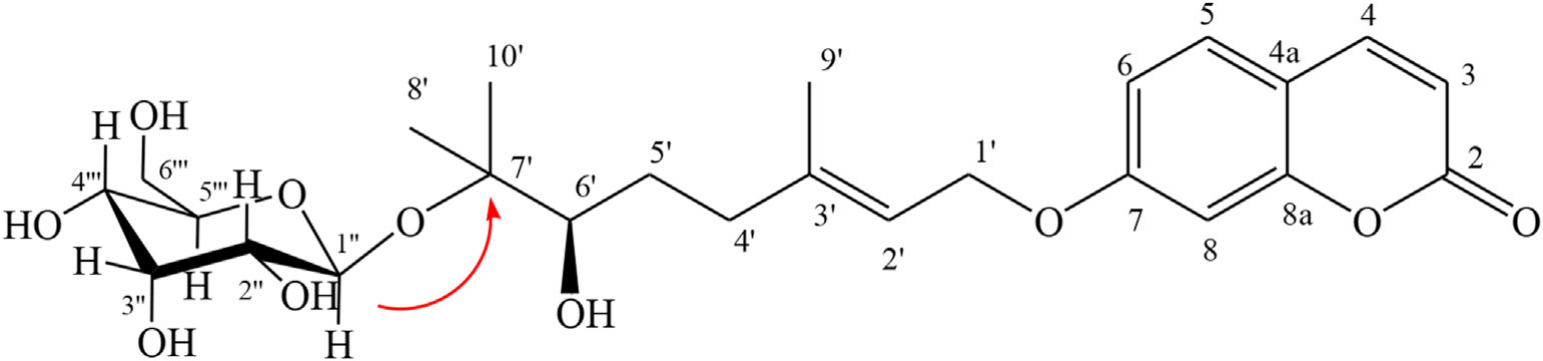
Relevant HMBC, (H C) correlations in Pituranthoside (2).

**Figure 5:**
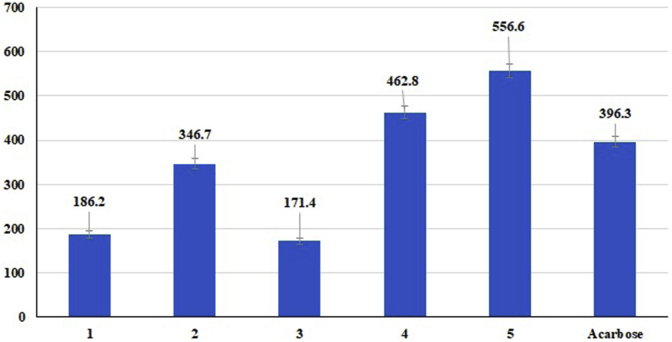
α-Glucosidase inhibitory activity of compounds 1–5.

**Figure 6:**
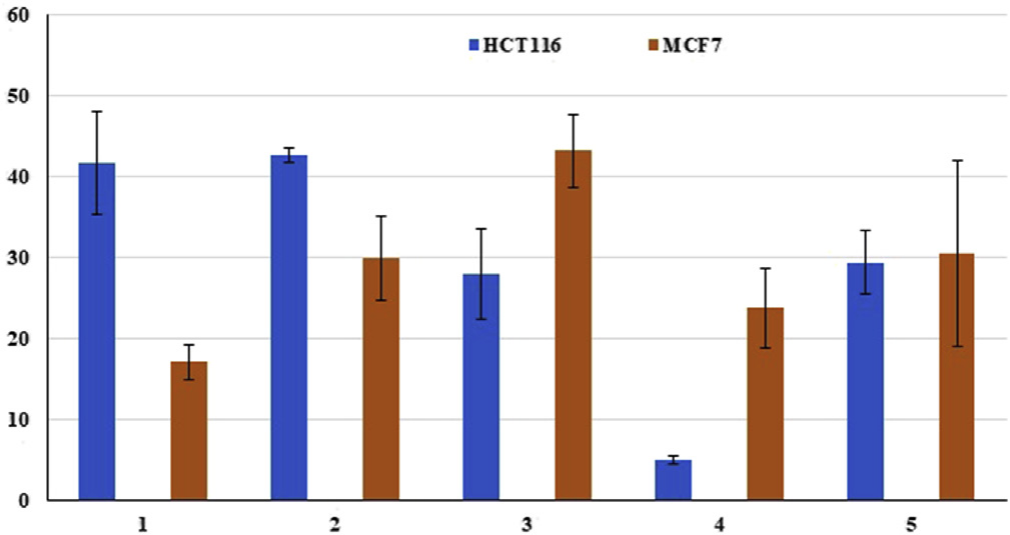
Cytotoxic activity of compounds 1–5.

**Figure 7:**
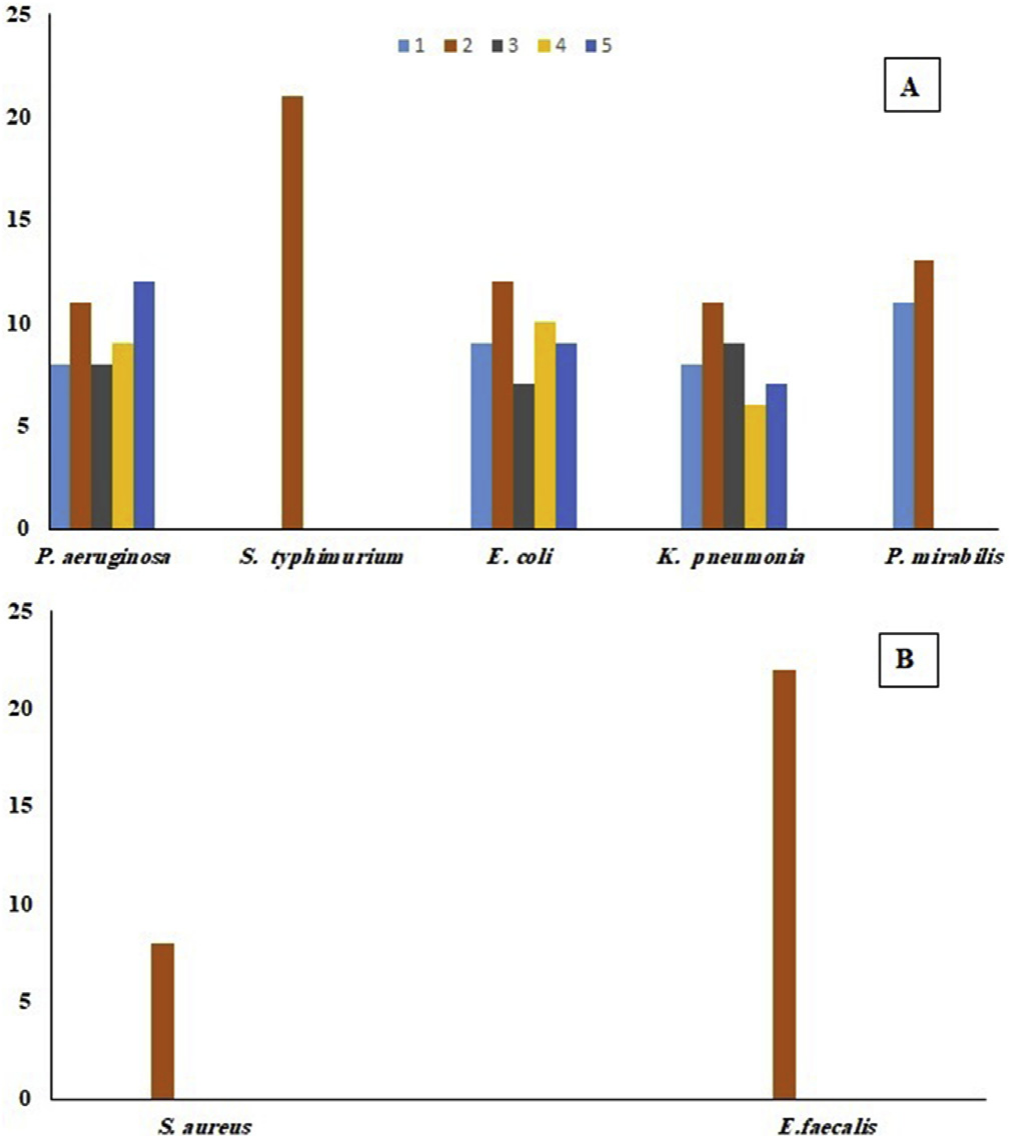
Antibacterial activity of compounds 1–5 against Gram-negative bacteria (a) and Gram-positive bacteria (b).

Was isolated as a white powder, its molecular formula, C_19_H_24_O_6_, was deduced by ESI-MS (positive-ion mode) with an ion peak at m/z 333.17 [M + H]^+^ (Figure 1S, Supplementary material). The IR spectrum (Figure 2S, Supplementary material) showed absorptions bands of hydroxyl (3473 cm^−1^), carbonyl (1724 cm-^1^), and aromatic ring groups (1610, 1507 and 1459 cm^−1^). The ^1^ H-NMR spectrum of **1** in CDCl_3_ (Table 1 and Figure 3S, Supplementary material) showed two *cis* olefinic protons at δ_H_ 6.26 (d, *J* = 9.5 Hz, H-3) and 7.66 (*d*, *J* = 9.5 Hz, H-4) and three aromatic protons at δ_H_ 7.38 (d, *J* = 8.5 Hz, H-5), δ 6.88 (dd, *J* = 8.5, 2.5 Hz, H-6), and δ_H_ 6.85 (d, *J* = 2.5 Hz, H-8), indicating a monosubstituted coumarin skeleton (Wang et al., 2020). Analysis of the ^13^C NMR and HSQC spectra (Table 1) and (Figure 4S and Figure 5S, Supplementary material) suggested a coumarin system with an additional C_10_ moiety including three methyl groups, two methylene groups, two olefin carbons, an oxygenated quaternary carbon an oxygenated methylene and an oxygenated methine groups and. The full assignments of the 1D-NMR spectra were confirmed by 2D-NMR (COSYand HMBC experiments) (Figure 6S and Figure 7S, Supplementary material). The correlations H-6’/H-5′, H-5’/H-4′ and H-1’/H-2′ indicated in the ^1^H–^1^H COSY spectra of **1** indicated the presence of two subunits [-CH_2_(4′)-CH_2_(5′)-CHOH(6′)] and [-CH(3′) = CH(2′)-CH_2_(1′)-] (Figure 3). In combination with the HMBC long-range ^2^*J* and ^3^*J* correlations from Me-8′ and Me-10′ to C-7′ and C-6′, and the correlations from Me-9 to C-4′, C-3′ and C-2′ revealed the presence of a dihydroxylated geranyl side-chain skeleton (Figure 3). In addition, the location of the latter side chain in coumarin at C-7 was demonstrated by the HMBC cross-peaks from H-1’ to C-7 via oxygen atoms.

**Table 1:** ^1^ H and^13^C NMR data (500 and 125 MHz, CDCl_3_, CD_3_OD) for compounds (1–2).

Position	1		2	
	^13^C	^1^H [δ, mult, J (Hz)]	^13^C	^1^H [δ, mult, J (Hz)]
1		-	-	-
2	161.3	-	162.0	-
3	112.4	6.26 (d, 9.5)	111.9	6.27 (d, 9.5)
4	143.4	7.66 (d, 9.5)	144.5	7.90 (d, 9.5)
4a	113.0	-	112.5	-
5	128.9	7.38 (d, 8.5)	129.0	7.54 (d, 8.5)
6	113.2	6.88 (dd, 2.5, 8.5)	113.5	6.966.88 (dd, 2.5, 8.5)
7	162.0	-	162.4	-
8	101.5	6.85 (d 2.5)	101.0	6.92 (d, 2.5)
8a	155.2	-	155.7	-
1′	65.4	4.63 (d, 6.5)	65.4	4.70 (d, 6.5)
2′	118.9	5.55 (t, 6.5)	119.1	5.54 (t, 6.5)
3′	142.2	-	141.6	-
4′	36.5	2.40 (m) 2.20 (m)	36.12	2.35(m) 2.18(m)
5′	29.4	1.66 (m) 1.50 (m)	28.8	1.71(m) 1.42(m)
6′	77.6	3.36 (dd, 2, 10.5)	76.7	3.45 (dd, 2, 11)
7′	73.3	-	80.2	-
8′	26.5	1.23 (s)	22.2 (s)	1.24 (s)
9′	23.2	1.19 (s)	20.1(s)	1.23 (s)
10′	16.8	1.80(s)	15.3 (s)	1.81 (s)
1″	-	-	97.1	4.48 (d,8)
2″	-	-	73.7	3.17 (t,7.5)
3″	-	-	76.2	3.35 (m)
4″	-	-	70.1	3.31(m)
5″	-	-	76.0	3.24(m)
6″	-	-	61.1	3.82 (dd, 2.5, 11.5) 3.65 (dd, 5.5, 12)

#### 3.1.2 Pituranthoside (2)

Was isolated as a white powder, its molecular formula C_27_H_34_O_11_, as deduced from NMR data (Table 1) and (Figures 8S-12S, Supplementary material) and the fragmentation observed in the ESI-MS spectrum (Figure 13S, Supplementary material). The latter showed fragments from the loss of a sugar unit at m/z 333.17 [(M + H)-163]^+^ and a fragment at m/z 181.09 attributable to the sugar unit. The IR spectrum (Figure 14S, Supplementary material) displayed absorptions of hydroxyl (3412 cm^−1^), carbonyl (1728 cm-^1^), and aromatic rings (1617, 1557 and 1427 cm^−1^). The NMR data (Table 1) of **2** displayed a high degree of similarity to those of **1**, except for the presence of the β-D-glucopyranose moiety in **2**. The significant shift of the carbon at δ_C_ 80.2 and the HMBC correlation from anomeric proton H-1″at δ_H_ 4.48 to oxygenated quaternary carbon C-7 at δ_C_ 80.2 suggested the location of β-D-glucopyranose in C-7 (Figure 4) and (Figure 12S, Supplementary material). Furthermore, the spectral data of products **1** and **2** (Table 1) compared with the literature (Halim et al., 1995) allowed us to confirm the proposed structures as well as the stereochemistry.

#### 3.1.3 Isoimperatorin (3)

White amorphous solid. Its molecular formula, C_16_H_14_O_4_, was deduced by ESI-MS (271.10 [M + H]^+^ (Figure 15S, Supplementary material), and NMR data (see Table 2) and (Figure 16S and Figure 17S, Supplementary material). IR (cmֿ ^1^, KBr): 3416, 3160, 2953, 1718,1605, 1479, 1426, 1171, 1070 (Figure 18S, Supplementary material). This compound was isolated previously from *Poncirus trifoliata* Raf (Pokharel et al., 2006). and *Deverra denudata* (syn. *Pituranthos chloranthus*) (Aloui et al., 2015).

**Table 2:** ^1^ H and^13^C NMR data (500 and 125 MHz, CDCl_3_) for compounds (3–5).

Position	3		4		5	
	^13^C	^1^H [δ, mult, J (Hz)]	^13^C	^1^H [δ, mult, J (Hz)]	^13^C	^1^H [δ, mult, J (Hz)]
1		-		-		-
2	161.5	-	161.4	-	160.5	-
3	112.6	6.27 (d, 9.5)	112.9	6.30 (d, 9.5)	113.3	6.31 (d, 9.5)
4	139.6	8.18 (d, 9.5)	139.4	8.18 (d, 9.5)	139.9	8.15 (d, 9.5)
4a	107.6	-	106.6	-	107.8	-
5	152.9	-	149.6	-	144.5	-
6	112.5	-	112.1	-	115.0	-
7	158.2	-	158.4	-	149.9	-
8	94.1	7.16 (s)	94.5	7.16 (s)	128.2	-
8a	149.1	-	153.0	-	143.7	-
2′	144.9	7.62 (d, 2.5)	144.9	7.62 (d, 2.0)	145.4	7.66 (d, 3.0)
3′	105.0	6.98 (d, 2.5)	104.9	7.07 (d, 2.0)	105.3	7.02 (d, 3.0)
5-OMe	-	-	60.2	4.29 (s)	61.9	4.20 (s)
8-OMe	-	-	-	-	61.5	4.19 (s)
1″	69.7	4.93 (d,7)	-	-	-	-
2″	119.2	5.66 (m)	-	-	-	-
3″	139.8	-	-	-	-	-
4″	26.1	1.83	-	-	-	-
5″	18.1	1.72	-	-	-	-

#### 3.1.4 Bergapten (4)

White amorphous solid. The molecular formula C_12_H_8_O_4_ was determined by ESI-MS (217.05 [M + H]^+^, Figure 19S, Supplementary material), NMR data (see Table 2) and (Figure 20S and Figure 21S, Supplementary material) and IR (cmֿ ^1^, KBr): 3414, 3142, 3113, 2959,1731, 1625, 1580, 1470, 1360, 1257, 1214, 1155 (Figure 22S, Supplementary material).

#### 3.1.5 Isopimpinellin (5)

White, amorphous solid. Its molecular formula, C_13_H_10_O_5_, was deduced by ESI-MS: 247 [M + H]^+^ (Figure 23S, Supplementary material), NMR data (see Table 2) and (Figure 24S and Figure 25S, Supplementary material) and IR (cmֿ ^1^, KBr): 3434, 2960, 1729, 1627,1603, 1581, 1456, 1386, 1325 (Figure 26S, Supplementary material).

Compounds **4** and **5** have been previously identified from many plant taxa, including *P*. *triradiatus* (Hochst ex Boiss.) (Halim et al., 1995), *Metrodorea mollis and Pilocarpus spicatus* (Rutaceae) (Madeiro et al., 2017).

### 3.2 α-Glucosidase inhibitory activity

The inhibitory abilities of α-glucosidase by the **5** isolated compounds, expressed as IC_50_ values, are shown in Figure 5. Among the 5 tested compounds, **1**, **2** and **3** displayed the best inhibitory activity, with IC_50_ values of 186.2 ± 8.3, 346.7 ± 11.2 and 171.4 ± 7.6 μM, respectively. Compounds **1**, **2** and **3** exhibited α-glucosidase inhibitory activity somewhat higher than that shown by acarbose (IC_50_ 396.3 ± 12.5 μM). When we compared the structure of linear furanocoumarins (**3**, **4** and **5**), isoamperatorin (3) contained a (CH_3_)_2_CHCH_2_O- group at the C-6 position, which seemed to be more potent against the α-glucosidase enzyme than Bergapten (**4**), which contains a methoxy group at the C-6 position, and isopimpinellin, with two methoxy groups at the C-6 and C-8 positions (Figure 1 and Figure 5). The structure-activity relationship in compounds **1** and **2** is the presence or absence of β-d-glactopyranoside at C-7’. In fact, the presence of a β-d-glactopyranoside group at C-7’ significantly decreased the activity. The α-glucosidase inhibitory activity (in vitro) of both marmin (**1**) and pituranthoside (**2**) was reported for the first time by the present investigation. Karakaya et al., (2018) reported that the α-glucosidase activity of some furanocoumarins isolated from *Ferulago bracteata* was in agreement with our results. Previous studies have shown that coumarins and furanocoumarins have anti-α-glucosidase activity, such as fraxetin from *Arcytophyllum thymifolium* (Milella et al., 2016), xanthotoxin from *Ferulago bracteata* roots (Karakaya et al., 2018), imperatorin from *Ducrosia anethifolia* (Shalaby et al., 2014) and bergapten and coumarin derivatives from fruits of *Pandanus tectorius* (Luo et al., 2012; Nguyen et al., 2016).

### 3.3 Cytotoxic activity

The cytotoxic activity of isolated compounds was tested (100 μM) against two different cancer cell lines: HCT-116 and MCF-7. Figure 6 shows the percentage of inhibition of cell lines induced by the 5 compounds. For MCF-7 cell line, the percentage of inhibition varied between 43.2% (compound **3**) and only 17.1 % (compound **1**). MCF-7 seems to be more sensitive to compound **3** with the highest inhibition percentage (43.2%). Furthermore, the cell line was reported to be sensitive to compounds **5** and **2** with inhibition percentages varying between 30.6% and 29.9%. However, compounds **1** and **2** seem to be more effective against HCT-116 cells cell line and showed inhibition percentages of 41.8% and 42.7% respectively. Moreover, the compounds **3** and **5** exhibited almost the same sensitivity against HCT-116 cell line. To the best of our knowledge, the cytotoxic activity of 3 compounds (**1**, **2**, and **3**) were not reported previously. In addition, the cytotoxic activity of the 2 monoterpenoid coumarins (1 and 2) and the furocoumarins (compounds 3) was reported for the first time. Compared with previous studies, the **4** compounds showed relatively low cytotoxic activity (Thanh et al., 2004; Sancho et al., 2004; Chauthe et al., 2015; Mottaghipisheh et al., 2018). Coumarin derivatives were described to possess several therapeutic applications such as antitumor therapy (Musa et al., 2008), in vivo and in vitro cytotoxic activity (Detsi et al., 2017; Oueslati et al., 2019).

### 3.4 Antibacterial activity

Figure 7 showed the antibacterial activities of compounds **1**–**5** (1 mg/mL). Both Gram-negative bacteria (Figure 7a) and Gram-positive bacteria (Figure 7b) were tested by the well diffusion method. The tested compounds revealed antibacterial activity, and the inhibition zones varied from 6 to 22 mm (Figure 7). Among the tested products, phellopterin (**2**) showed the highest inhibition zone values against the 7 tested bacterial strains (Gram-negative *S. typhimurium* with IZ = 21 mm and Gram-positive *E*. *faecalis* IZ = 22 mm). In addition, compound **2** had a greater activity than compound 1; these results can be clarified by the presence of β-glcopyranose at C-7’ position. However, this is the first study on the antibacterial activity of pituranthoside (**2**) investigated in this study. In addition, previous studies have shown the antibacterial activity of marmin (**1**) against different bacterial strains (Tsassi et al., 2010). Our results are similar to those of previous studies conducted on related marmin (**1**) against *E*. *coli* with 9.5 mm as the inhibition zone. Furanocoumarins (**3**, **4** and **5**) showed similar effects against 3 bacterial strains (*P*. *aeruginosa*, *E*. *coli* and *K*. *pneumonia*), with inhibition zone values ranging from 6 to 12 mm. However, these compounds did not seem to be active against 4 bacterial strains (*S*. *typhimurium*, *K*. *pneumonia*, *E*. *faecalis* and *P*. *mirabilis*). The antimicrobial properties of several furanocoumarins have been reported previously, for Bergapten isolated from *Treculia obovoidea* (Kuete et al., 2007) and for Isoimperatorin isolated from the roots and fruits of *Ferulago trifida* Boiss (Tavakoli et al., 2018). and isopimpinellin isolated from *Prangos uloptera* roots (Razavi et al., 2009) and from *Peucedanum zenkeri* seeds (Ngwendson et al., 2003).

## 4 Conclusion

This phytochemical investigation of the n-hexane and ethyl acetate extracts of the seeds of *D. tortuosa* introduces the species as a source of marmin and furanocoumarin derivatives (**1**–**5**). Significant α-glucosidase inhibition was observed for compounds **1**, **2** and **3**, making them candidate phytochemicals for the development of new antidiabetic agents. Moreover, the high antibacterial activity of compounds **1** (Marmin) and **2** (Pituranthoside) against bacterial strains can be a good alternative for further antibacterial investigation.

## Declarations

### Author contribution statement

Mohamed Habib Oueslati, Arbi Guetat: Conceived and designed the experiments; Performed the experiments; Analyzed and interpreted the data; Contributed reagents, materials, analysis tools or data; Wrote the paper.

Jalloul Bouajila: Performed the experiments; Analyzed and interpreted the data; Wrote the paper.

A. Khuzaim Alzahrani, Jamith Basha: Performed the experiments; Contributed reagents; materials; analysis tools or data; Wrote the paper.

### Funding statement

The authors gratefully acknowledge the approval and the support of this research study by the grant n° 7664-SCI-2018-3-9-F. from the Deanship of Scientific Research at Northern Border University, Arar, Kingdom of Saudi Arabia.

### Data Availability Statement

Data included in article/supplementary material/referenced in article.

### Declaration of interests statement

The authors declare no conflict of interest.

### Additional information

No additional information is available for this paper.

## Appendix A Supplementary data

Supplemantary_material Supplemantary_material
