# Supplementary material for: Deverra tortuosa (Desf.) DC from Saudi Arabia as a new source of marmin and furanocoumarins derivatives with α-glucosidase, antibacterial and cytotoxic activities
Source: Heliyon. 2021 Apr 5;7(4):e06656. doi: 10.1016/j.heliyon.2021.e06656 (PMC8056227; doi:10.1016/j.heliyon.2021.e06656)

**SUPPLEMENTARY MATERIAL**

***Deverra tortuosa* (Desf.) DC. as a new source of marmin and furanocoumarins derivatives with α-glucosidase, antibacterial and cytotoxic activities**

**Mohamed Habib Oueslati^1,2*^, Arbi Guetat^3,4^, Jalloul Bouajila^5^, A. Khuzaim Alzahrani^6^, Jamith Basha^6^**.

**^1^**Northern Border University, College of Sciences, Department of Chemistry, Saudi Arabia.

^2^University of Carthage, Faculty of Science Bizerte, Laboratory of Hetero-organic compounds and Nanostructured Materials, Zarzouna, Tunisia,

**^3^**Northern Border University, College of Sciences, Department of Biological Sciences, Saudi Arabia.

**^4^**University of Carthage, National Institute of Applied Science and Technology, Department of Biology, Laboratory of Plant Biotechnology, B.P. 676, 1080 Tunis Cedex, Tunisia.

^5^University of Toulouse, Faculty of Pharmacy of Toulouse, Laboratory of IMRCP UMR CNRS, Toulouse F-31062, France,

^6^Northern Border University College of Applied Medicine, Department of Microbiology, P.O.Box 1321, 91431 Arar, Saudi Arabia.

**ABSTRACT**

*Deverra tortuosa* (Desf.) DC (Syn. *Pituranthos* *tortusus* (Desf.) Benth. & Hook.f. ex Asch. & Schweinf. is desert aromatic shrub widespread in the Northern Region of Saudi Arabia. In the framework of the present study, *n*-Hexane and ethyl acetate extracts of seed were fractionated in order to purify bioactive secondary metabolites. Five compounds were isolated for the first time from the seeds of the *D. tortuosa.*: Marmin 1 (1.57%), Pituranthoside 2 (0.145%), Isoimperatorin 3 (0.051%), Bergapten 4 (0.45%) and Isopimpinellin 5 (0.25%). Their structures were determined using extensive 1D and 2D NMR, (ESI)-HRMS and IR spectroscopic analyses and by comparison with literature data. The cytotoxic, α-Glucosidase and antibacterial activities effect of the pure phytochemicals are also evaluated.

**Key words:** *Deverra tortuosa*; Marmin; Furanocoumarins; cytotoxic activity; α‑glucosidase inhibition; Antibacterial activities

**List of figures**

**Figure 1S. ESI-MS spectrum of (1)**

**Figure 2S. IR spectrum of (1)**

**Figure 3S. ^1^H NMR spectrum of (1) in CDCl_3_**

**Figure 4S. ^13^C NMR spectrum of (1) in CDCl_3_**

**Figure 5S. ^1^H-^1^H COSY spectrum of (1) in CDCl_3_**

**Figure 6S. HSQC spectrum of (1) in CDCl_3_**

**Figure 7S. HMBC spectrum of (1) in CDCl_3_**

**Figure 8S. ^1^H NMR spectrum of (2) in CD_3_OD**

**Figure 9S. ^13^C NMR spectrum of (2) in CD_3_OD**

**Figure 10S. ^1^H-^1^H COSY spectrum of (2) in CD_3_OD**

**Figure 11S. HSQC spectrum of (2) in CD_3_OD**

**Figure 12S. HMBC spectrum of (2) in CD_3_OD**

**Figure 13S. ESI-MS spectrum of (2)**

**Figure 14S. IR spectrum of (2)**

**Figure 15S. ESI-MS spectrum of (3)**

**Figure 16S. ^1^H NMR spectrum of (3) in CDCl_3_**

**Figure 17S. ^13^C NMR spectrum of (3) in CDCl_3_**

**Figure 18S. IR spectrum of (3)**

**Figure 19S. ESI-MS spectrum of (4)**

**Figure 20S. ^1^H NMR spectrum of (4) in CDCl_3_**

**Figure 21S. ^13^C NMR spectrum of (4) in CDCl_3_**

**Figure 22S. IR spectrum of (4)**

**Figure 23S. ESI-MS spectrum of (5)**

**Figure 24S. ^1^H NMR spectrum of (5) in CDCl_3_**

**Figure 25S. ^13^C NMR spectrum of (5) in CDCl_3_**

**Figure 26S. IR spectrum of (5)**

**Fig. 1S. ESI-MS spectrum of (1)**


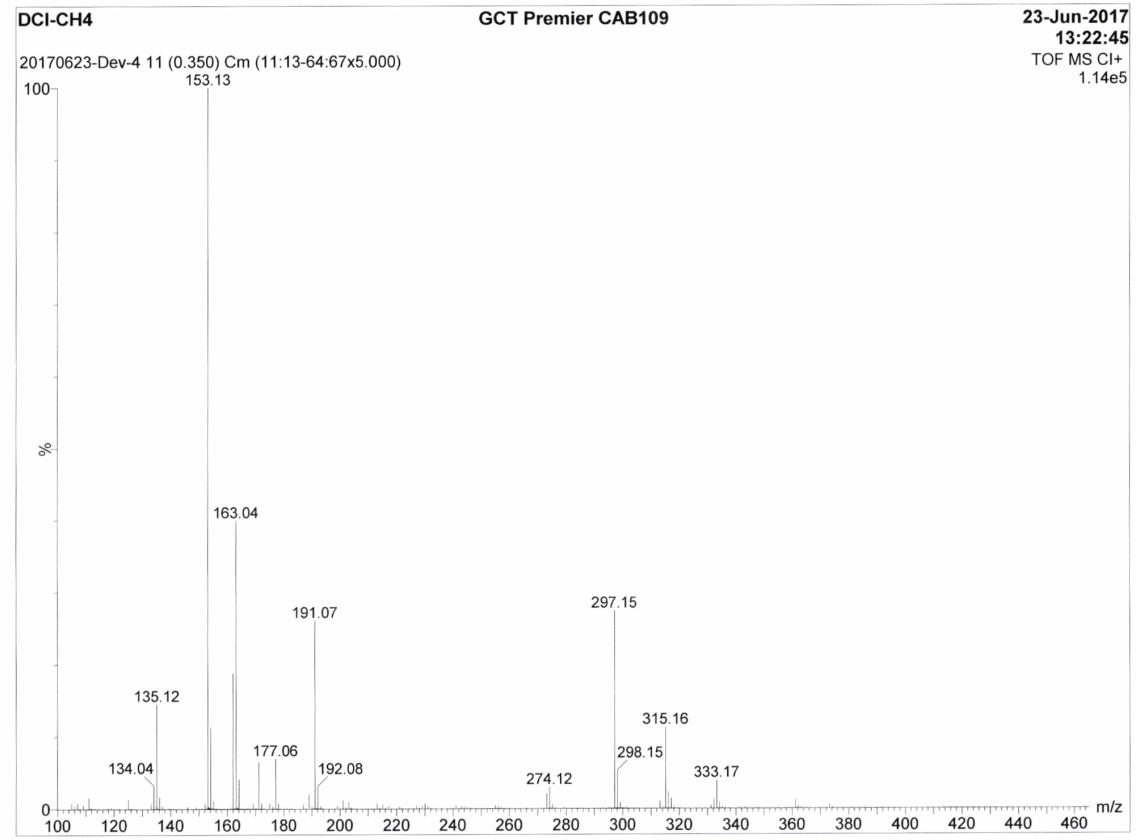


**Fig. 2S. IR spectrum of (1)**


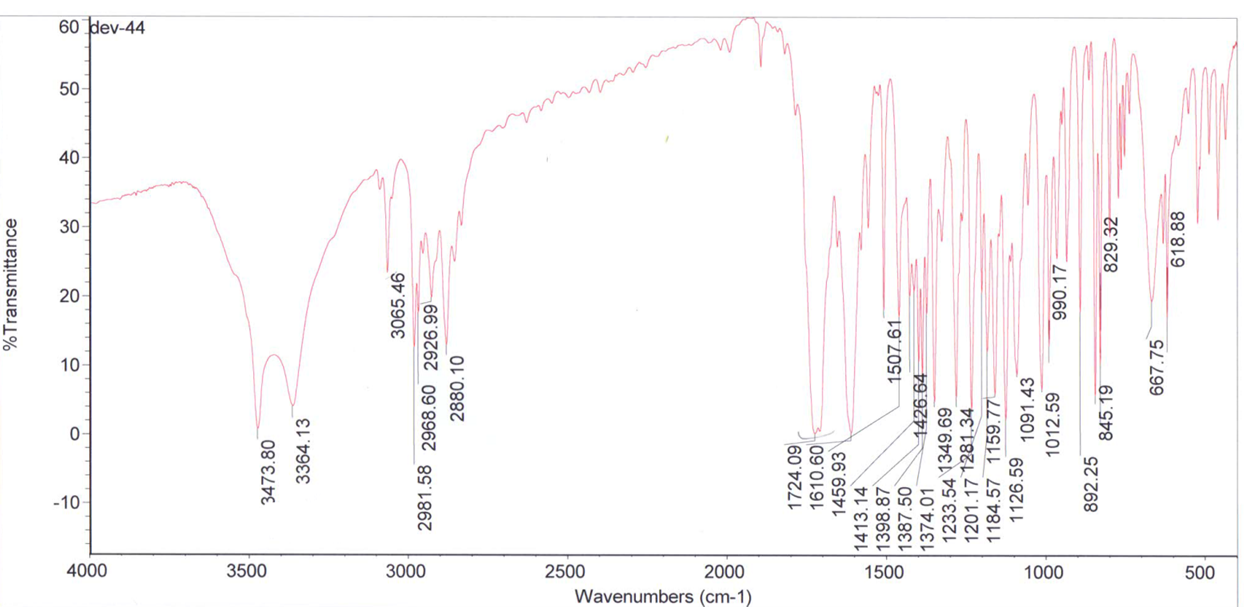


**Fig 3S. ^1^H NMR spectrum of (1) in CDCl_3_**

**
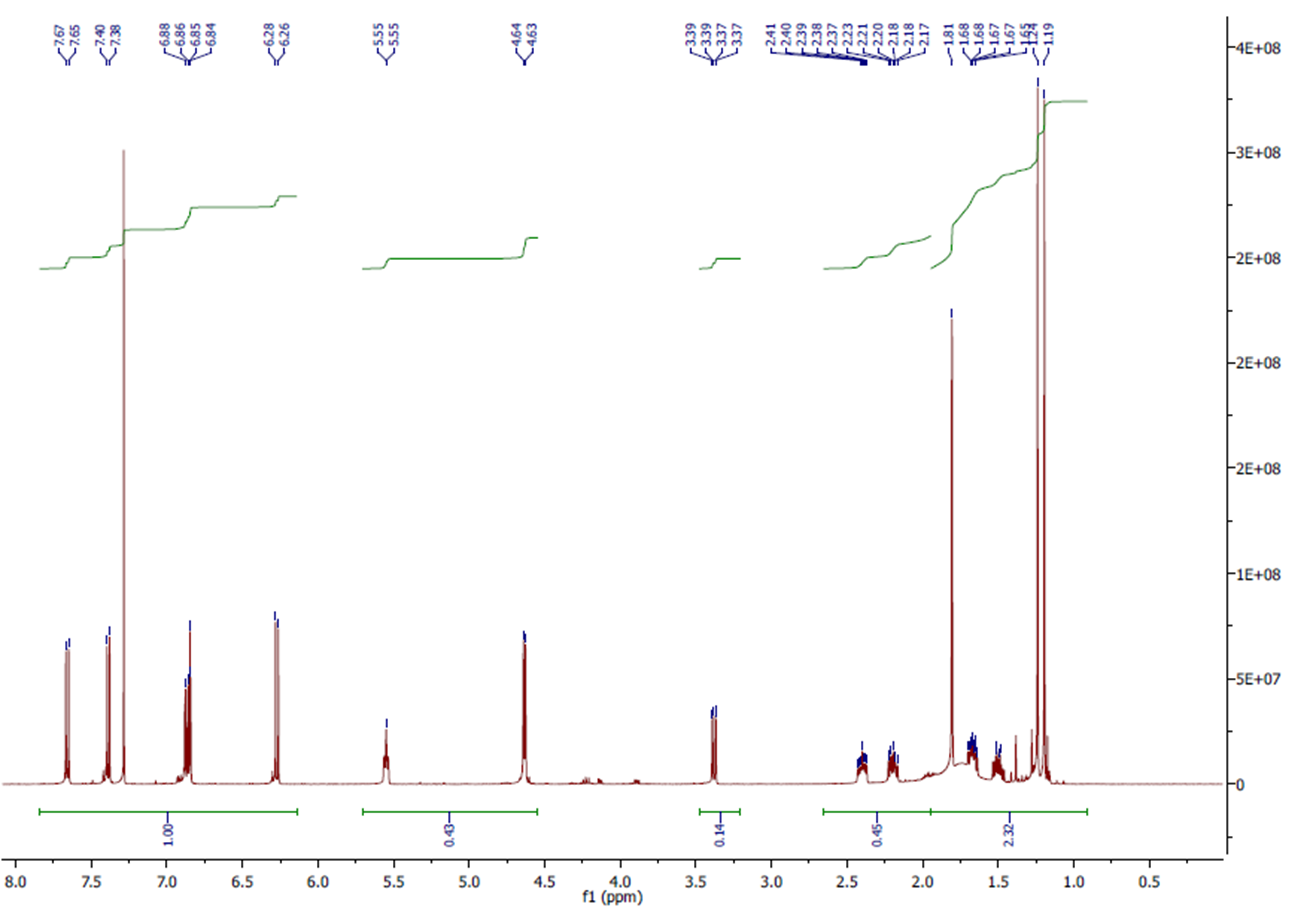
**

**Fig 4S. ^13^C NMR spectrum of (1) in CDCl_3_**

**
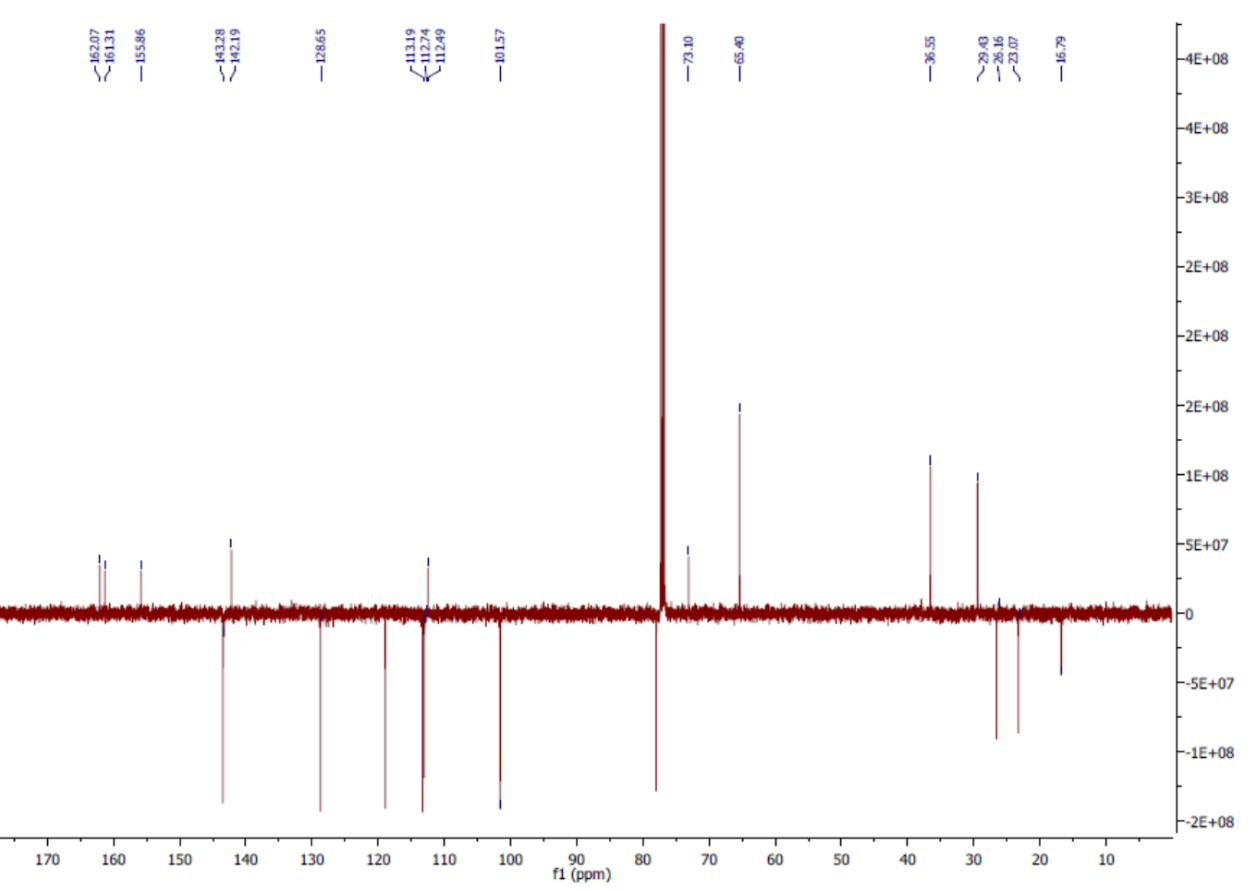
**

**Fig 5S. HSQC spectrum of (1) in CDCl_3_**

**
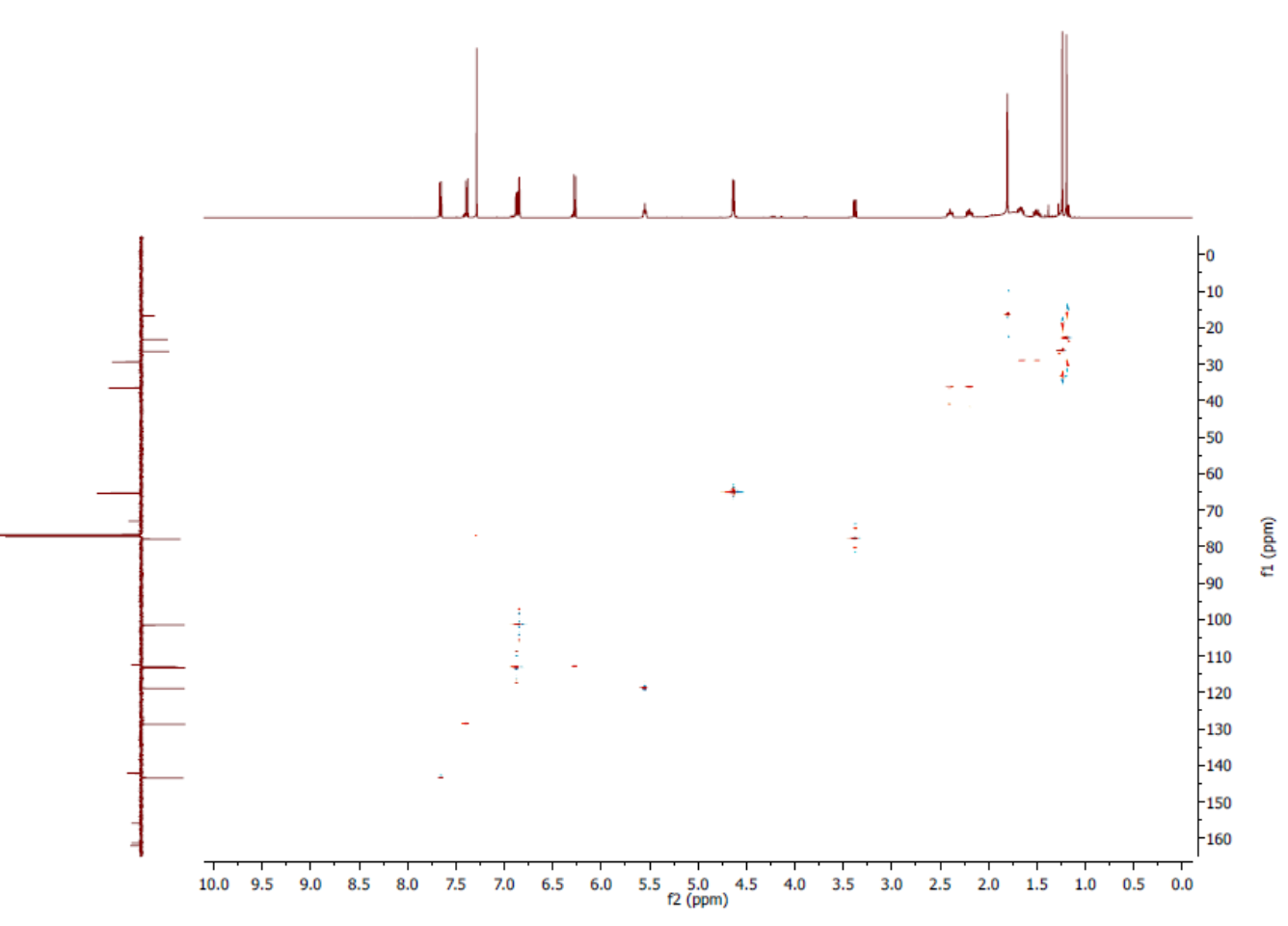
**

**Fig 6S. ^1^H-^1^H COSY spectrum of (1) in CDCl_3_**

**
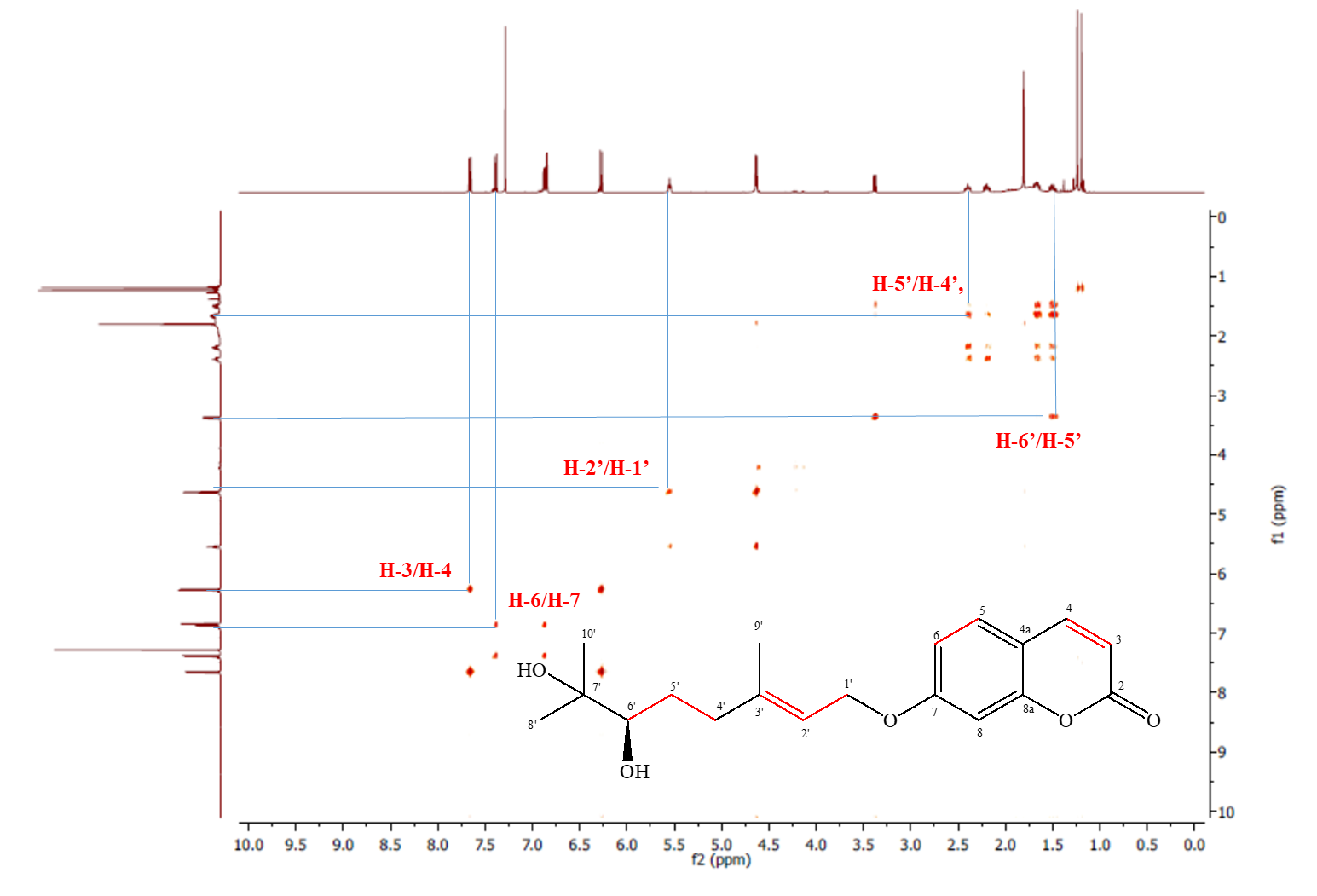
**

**Fig 7S. HMBC spectrum of (1) in CDCl_3_**

**
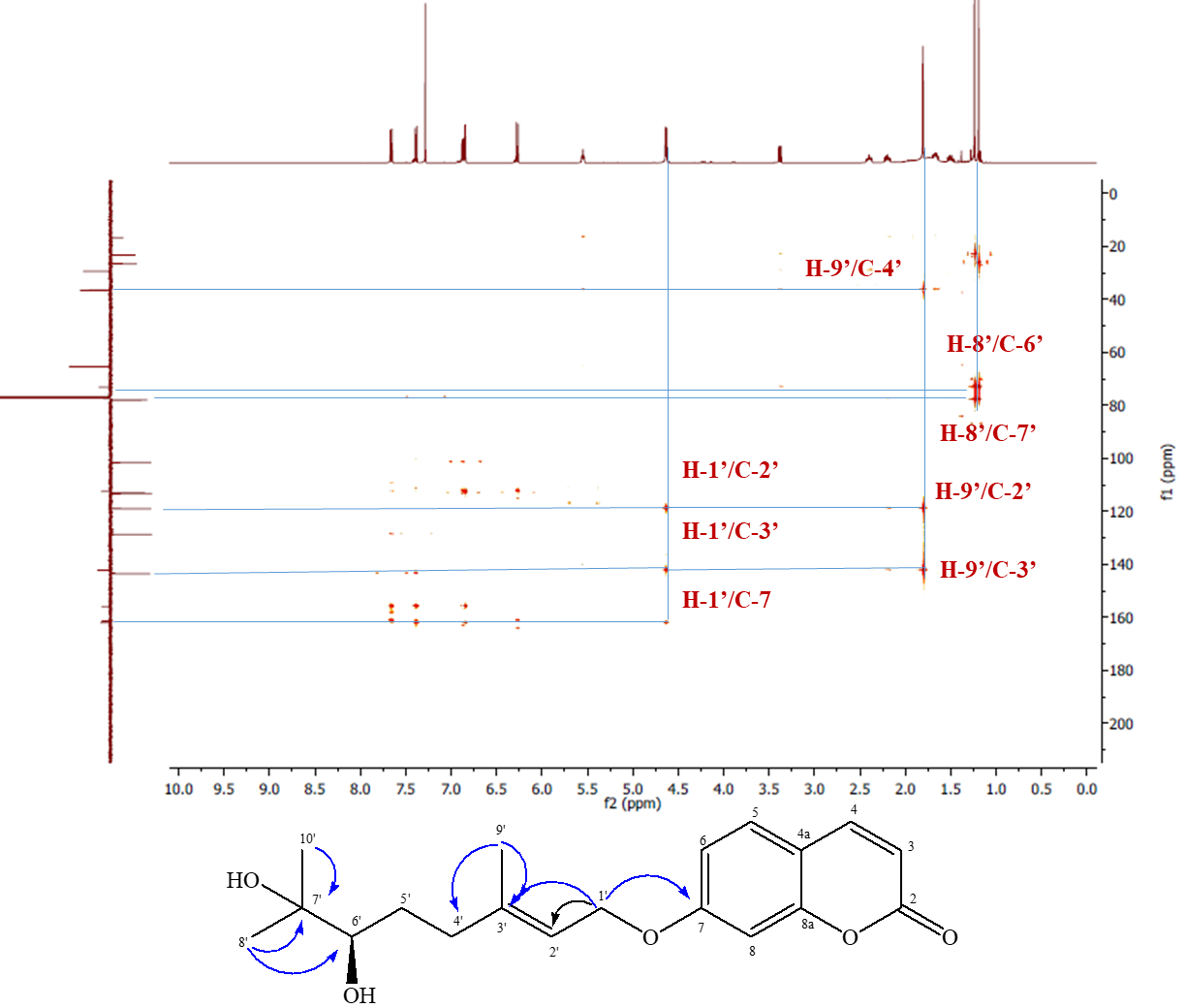
**

**Fig 8S. ^1^H NMR spectrum of (2) in CD_3_OD**

**
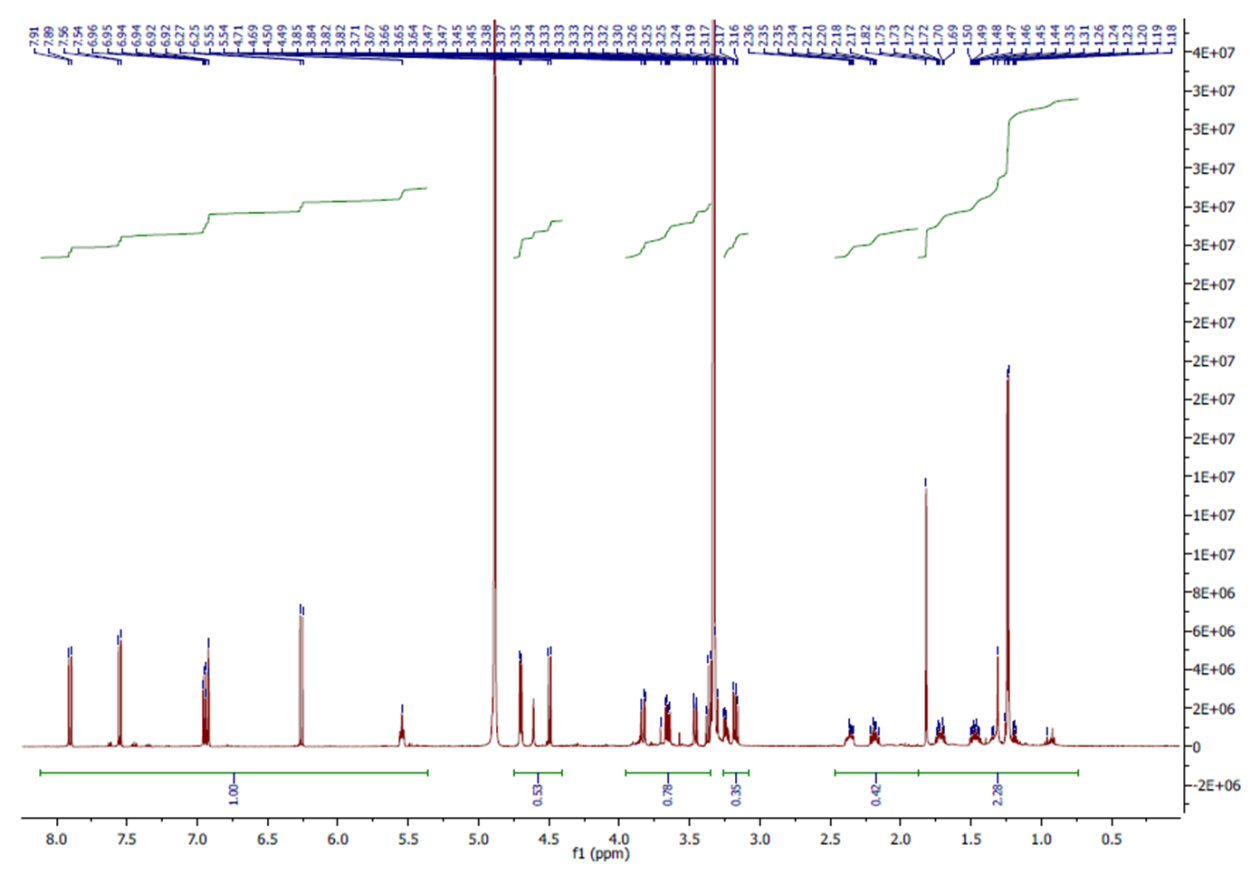
**

**Fig 9S. ^13^C NMR spectrum of (2) in CD_3_OD**

**
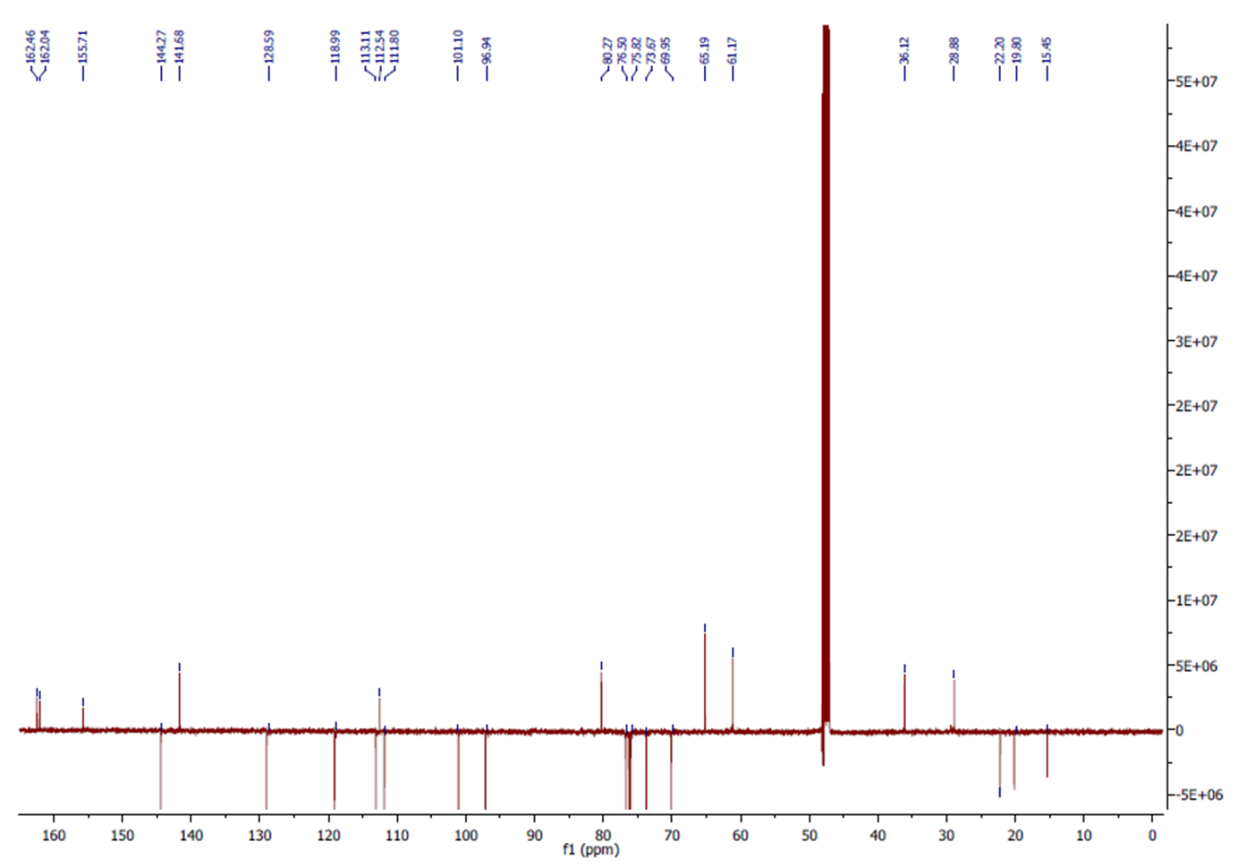
**

**Fig 10S. ^1^H-^1^H COSY spectrum of (2) in CD_3_OD**

**
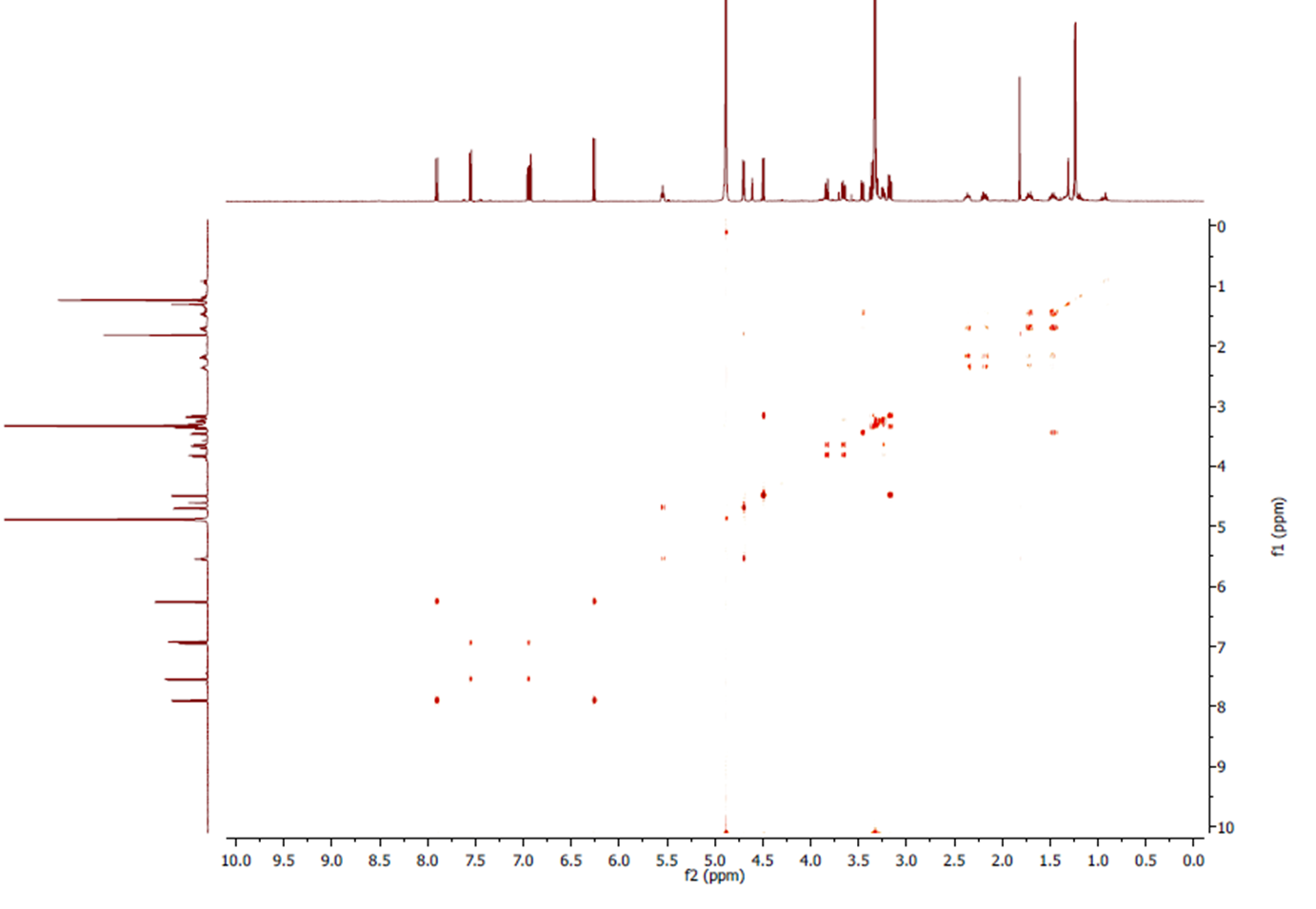
**

**Fig 11S. HSQC spectrum of (2) in CD_3_OD**

**
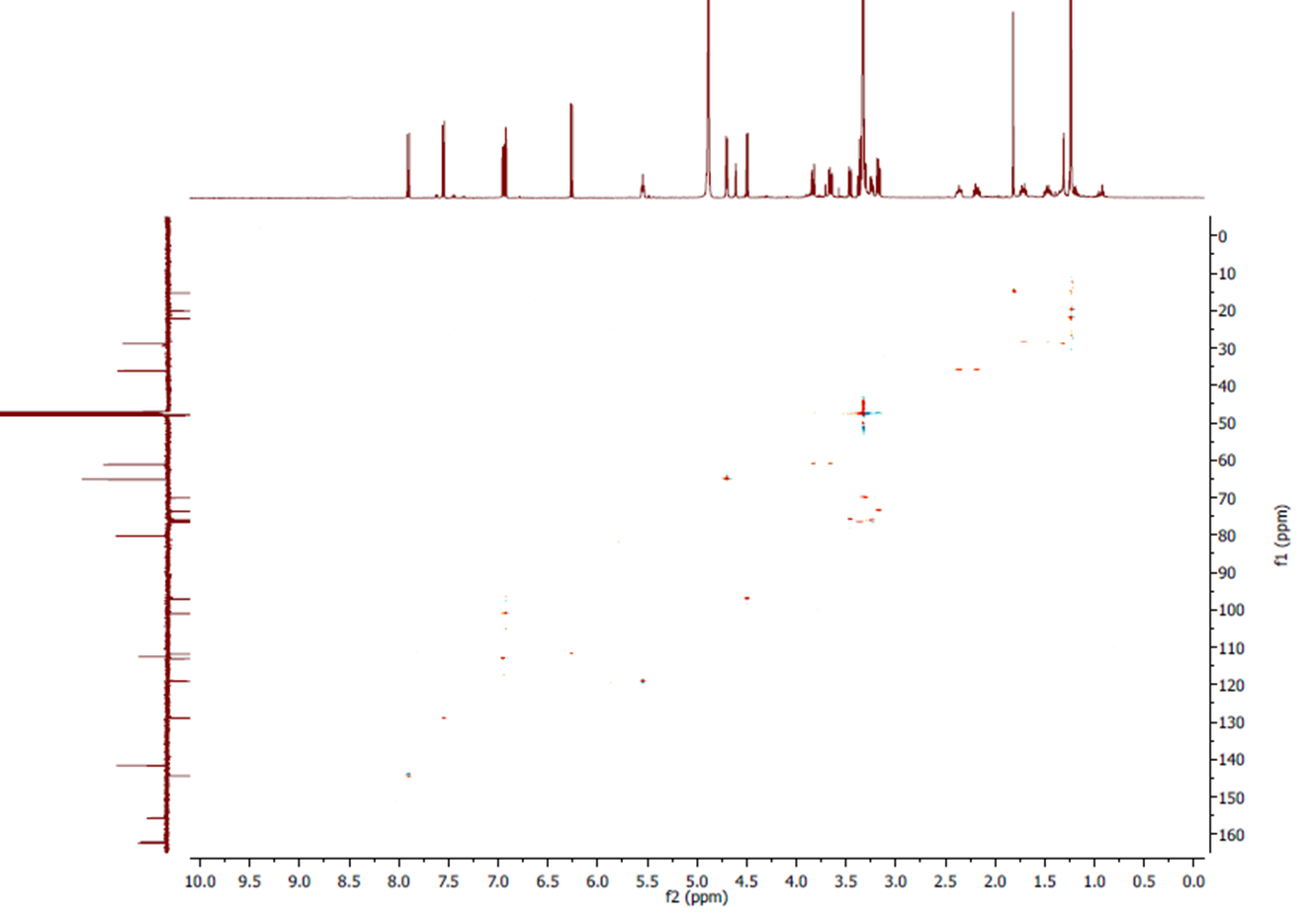
**

**Fig 12S. HMBC spectrum of (2) in CD_3_OD**

**
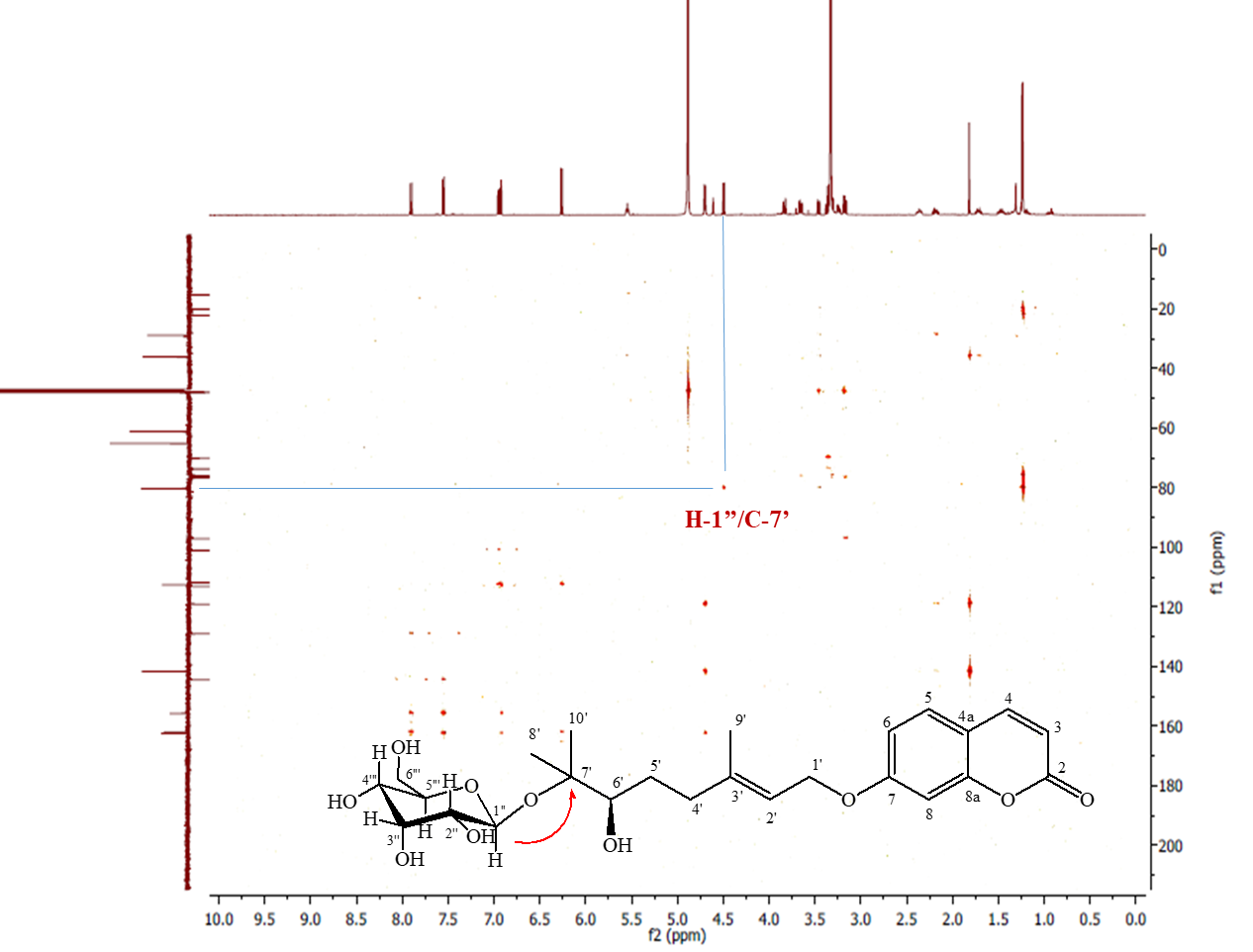
**

**Fig 13S. ESI-MS spectrum of (2)**

**
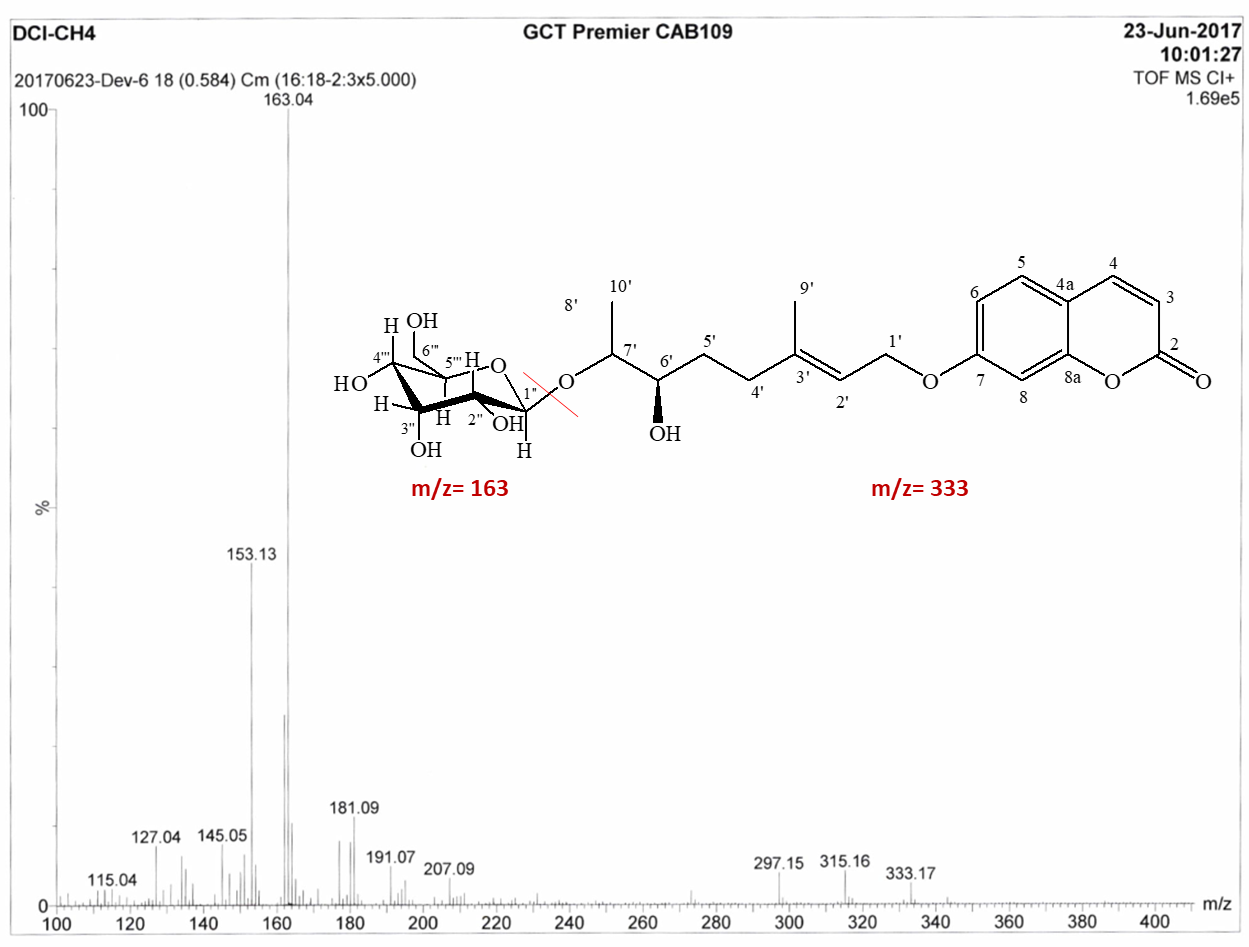
**

**Fig 14S. IR spectrum of (2)**


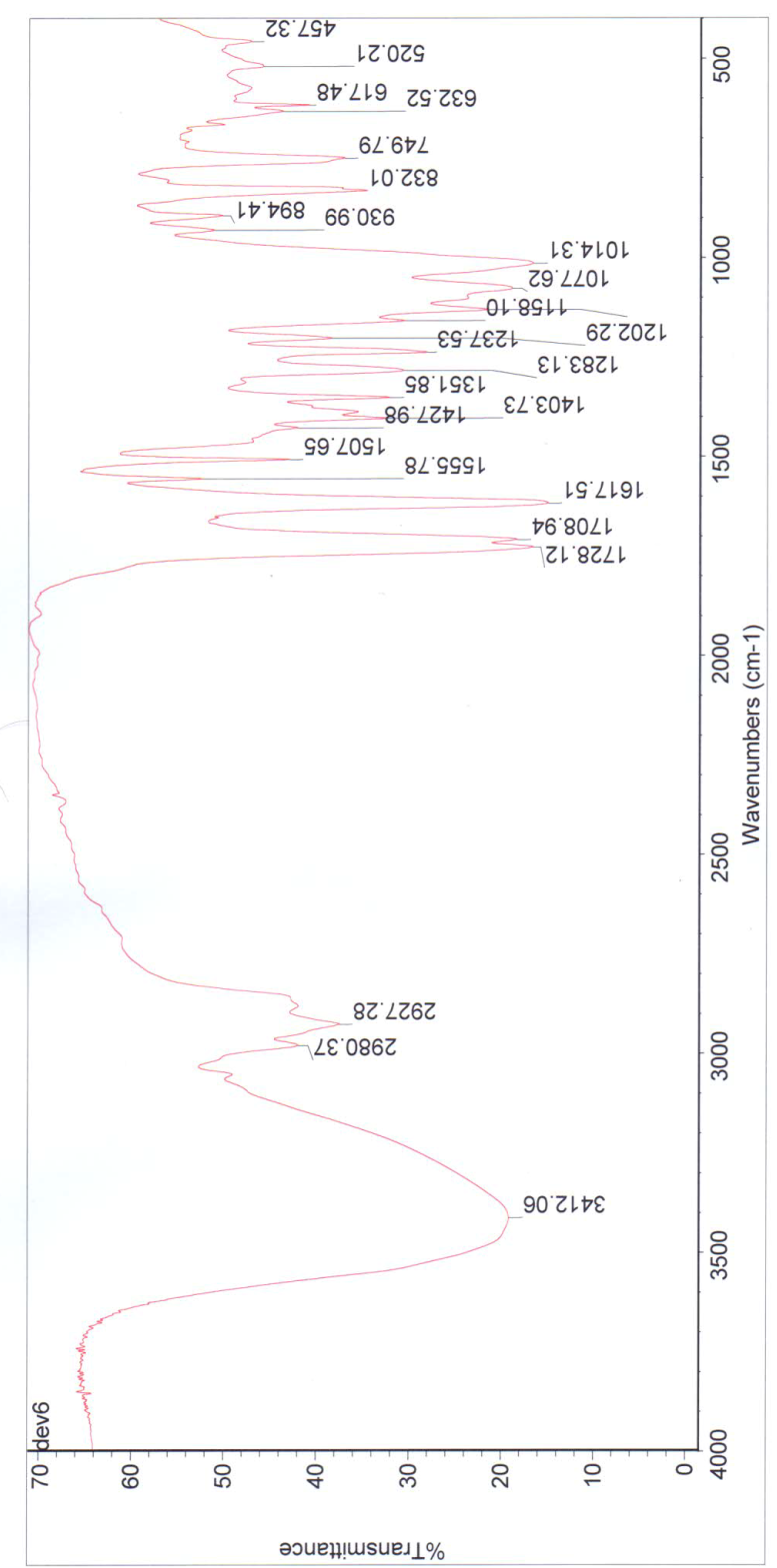


**Fig 15S. ESI-MS spectrum of (3)**


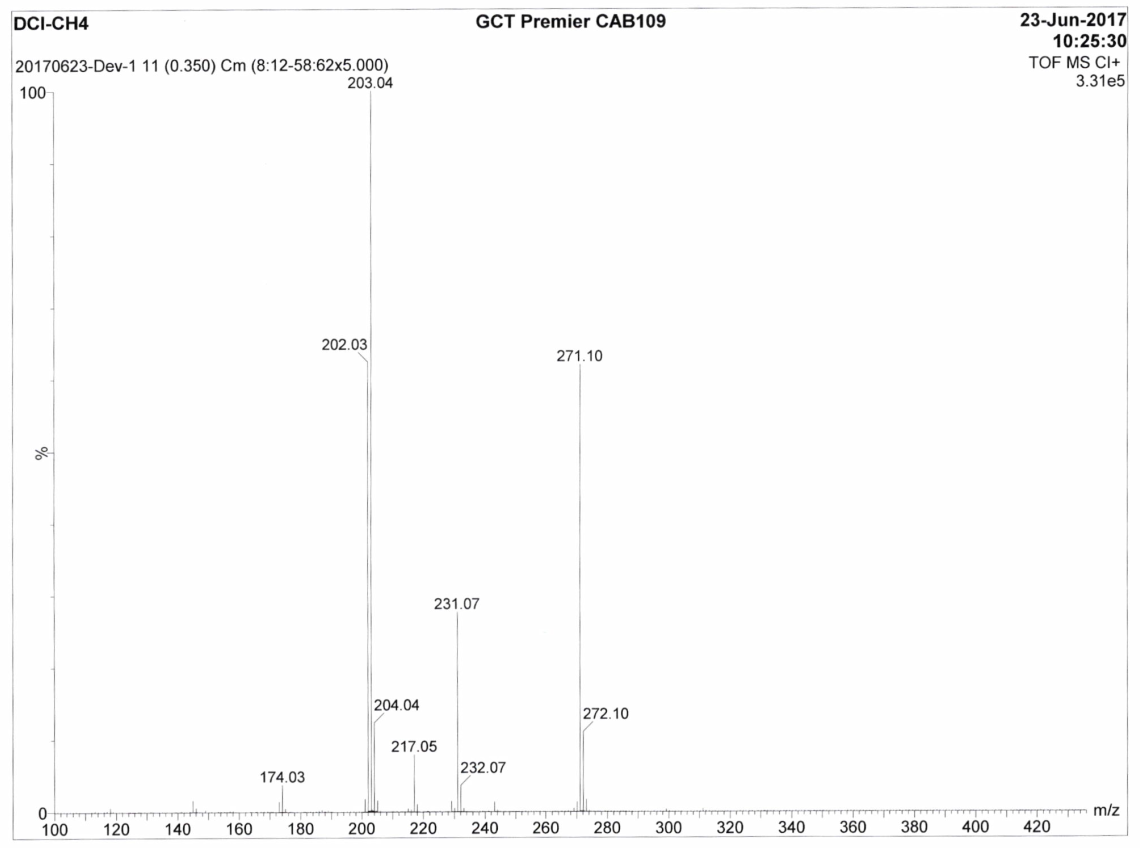


**Fig 16S. ^1^H NMR spectrum of (3) in CDCl_3_**

**
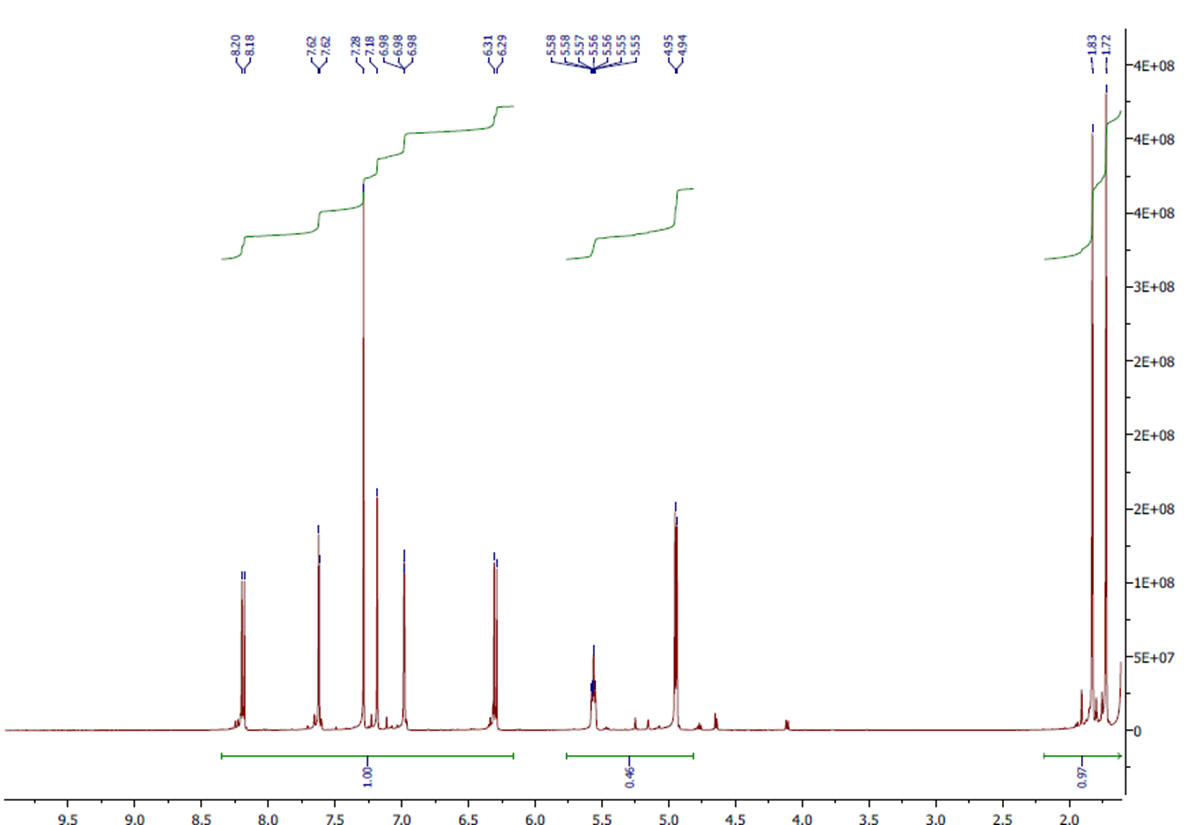
**

**Fig 17S. ^13^C NMR spectrum of (3) in CDCl_3_**

**
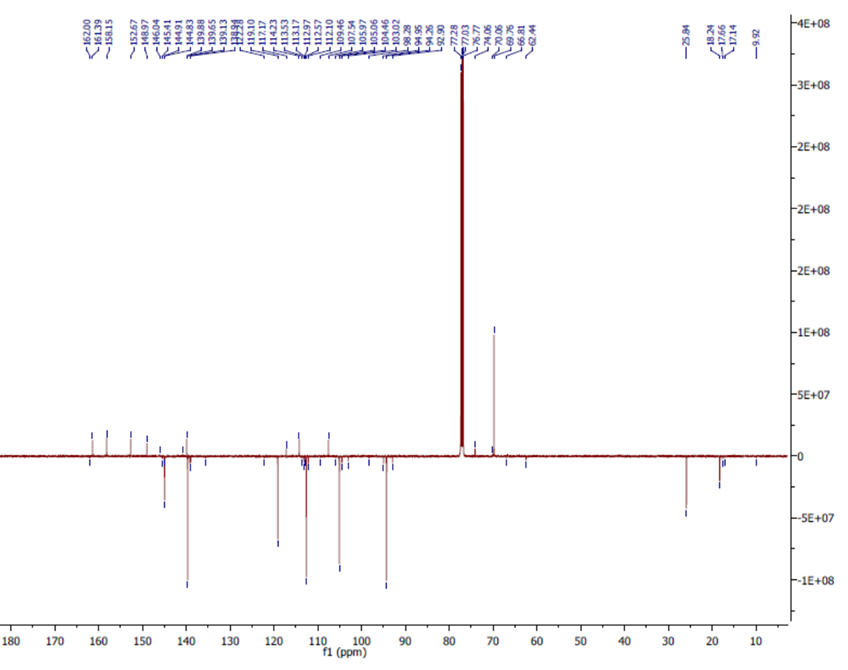
**

**Fig 18S. IR spectrum of (3)**

**
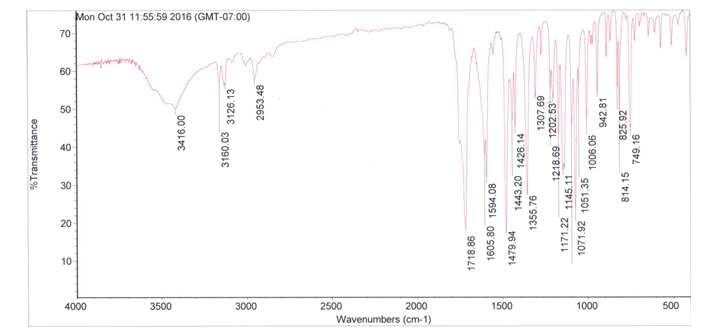
**

**Fig 19S. ESI-MS spectrum of (4)**


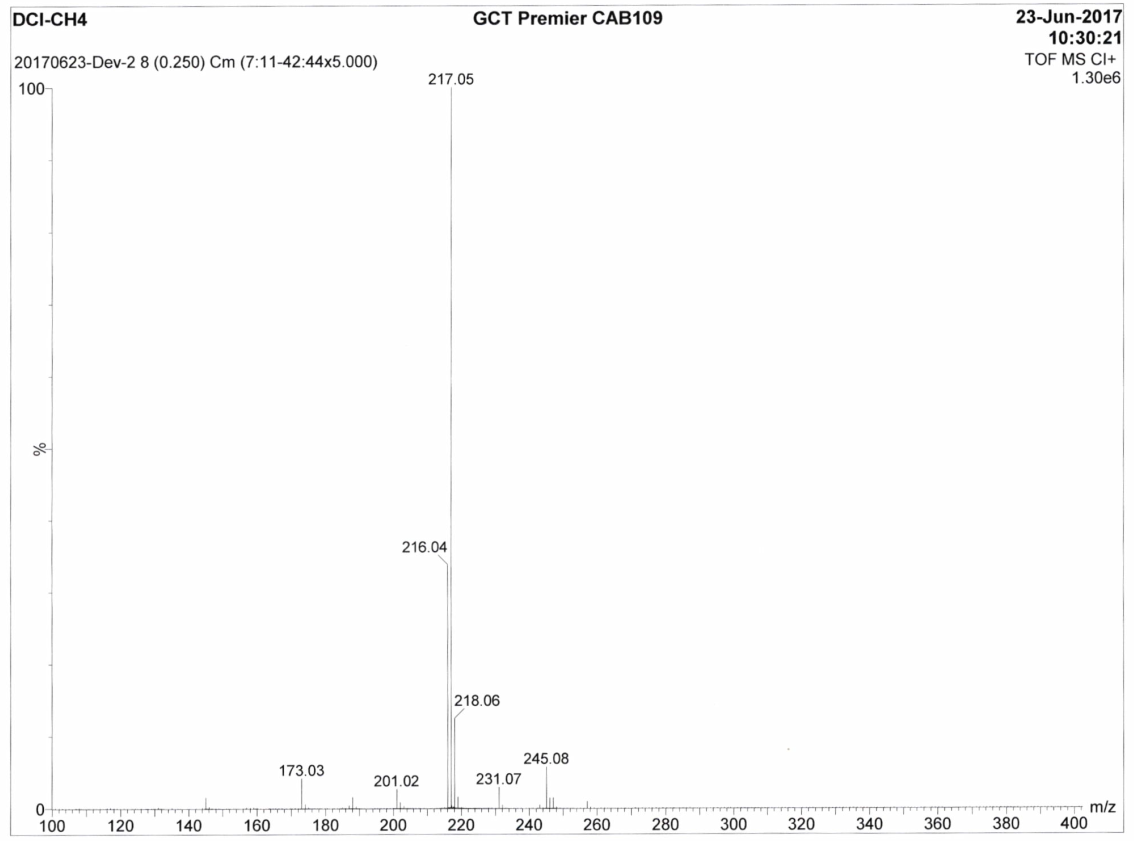


**Fig 20S. ^1^H NMR spectrum of (4) in CDCl_3_**

**
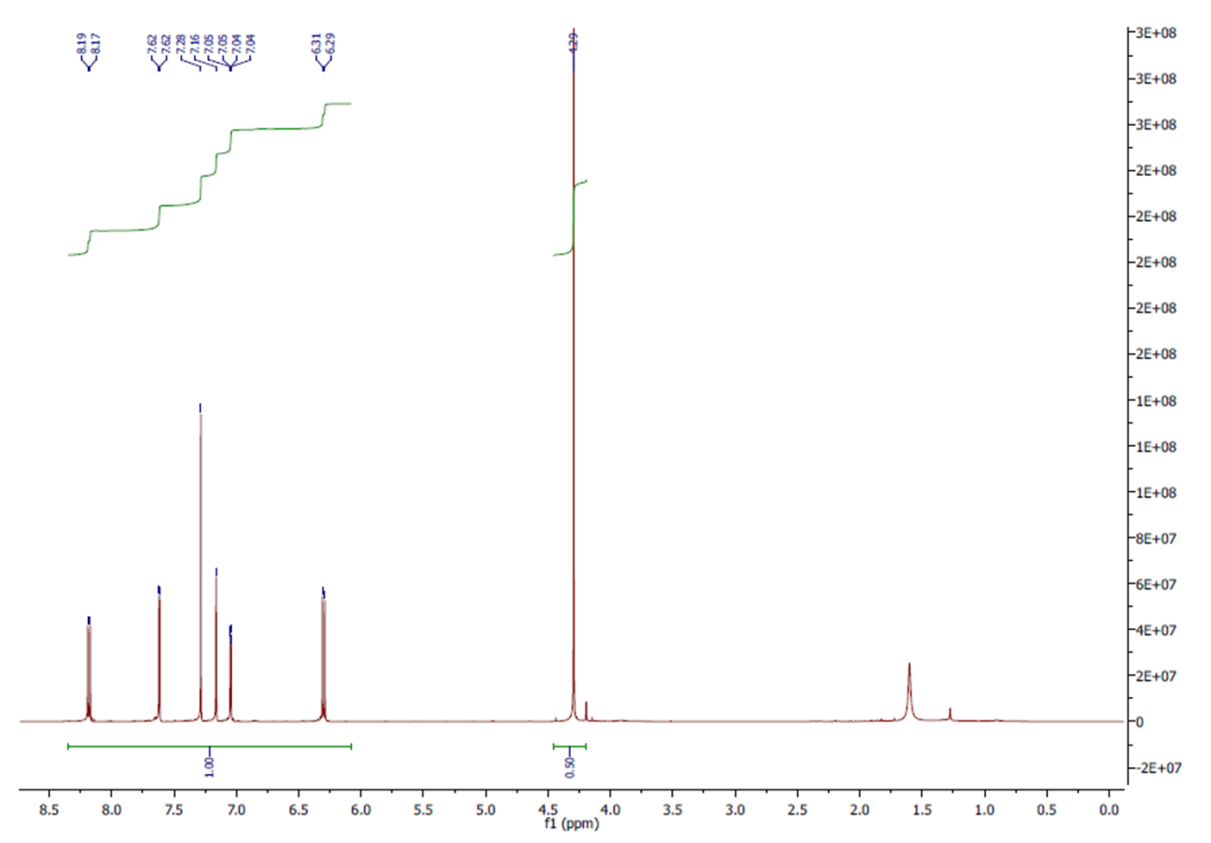
**

**Fig 21S. ^13^C NMR spectrum of (4) in CDCl_3_**

**
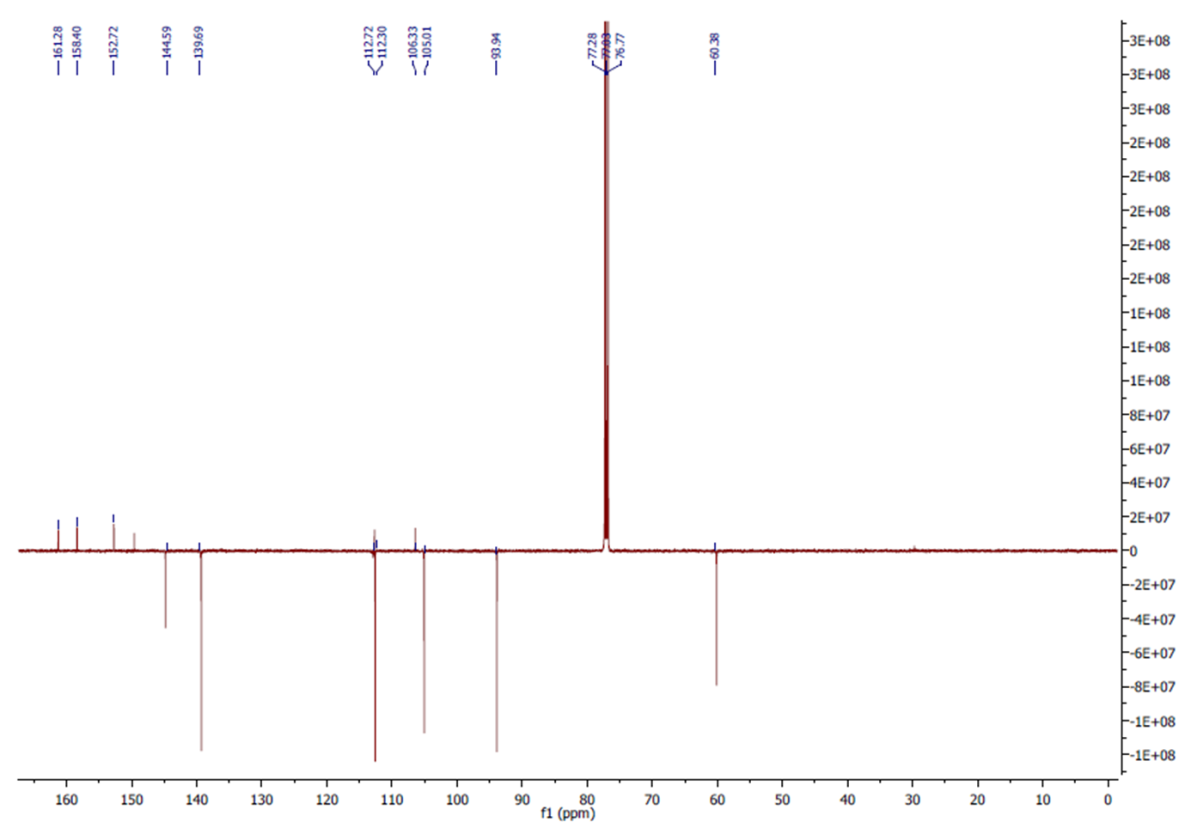
**

**Fig 22S. IR spectrum of (4)**


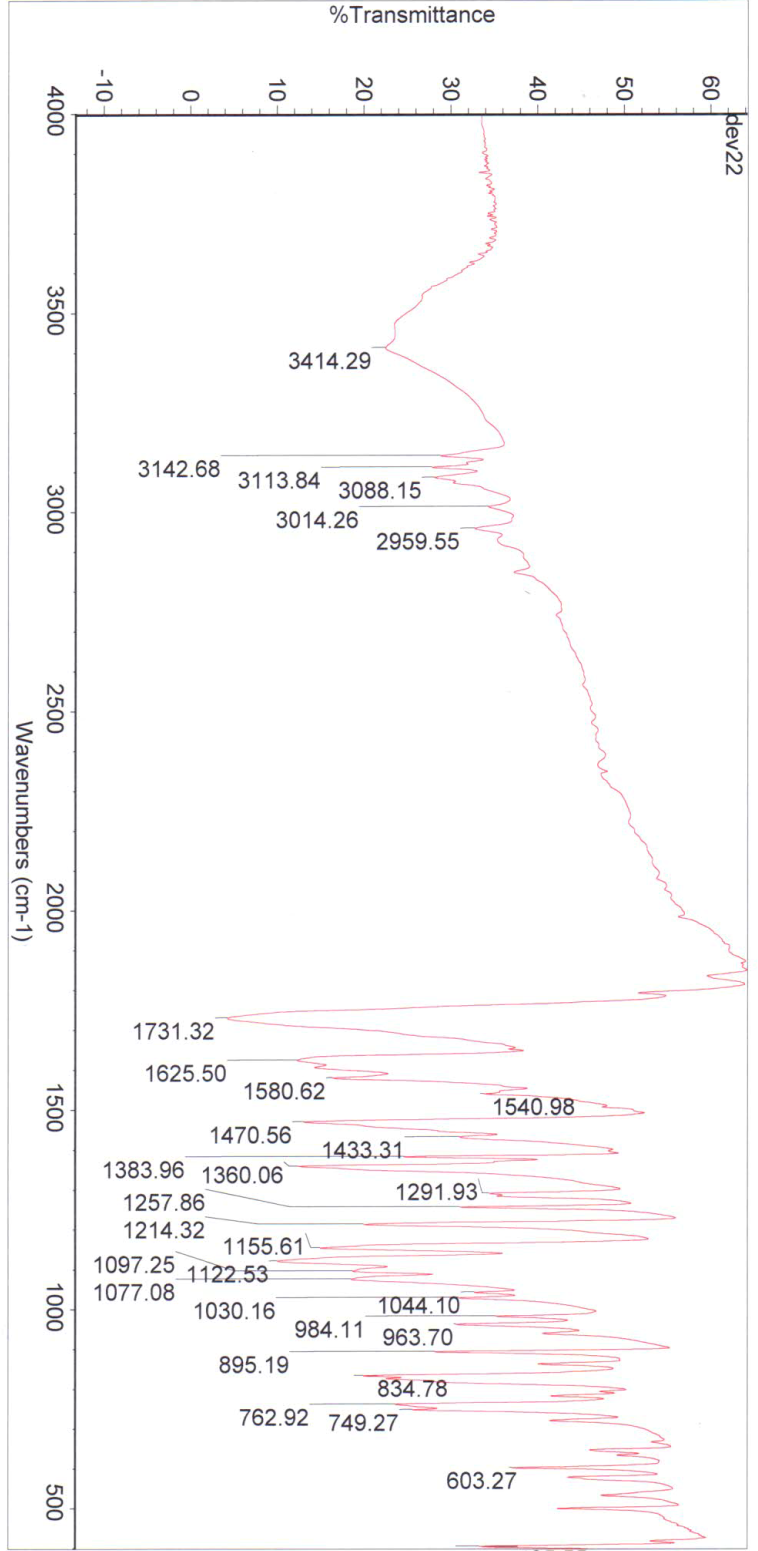


**Fig 23S. ESI-MS spectrum of (5)**


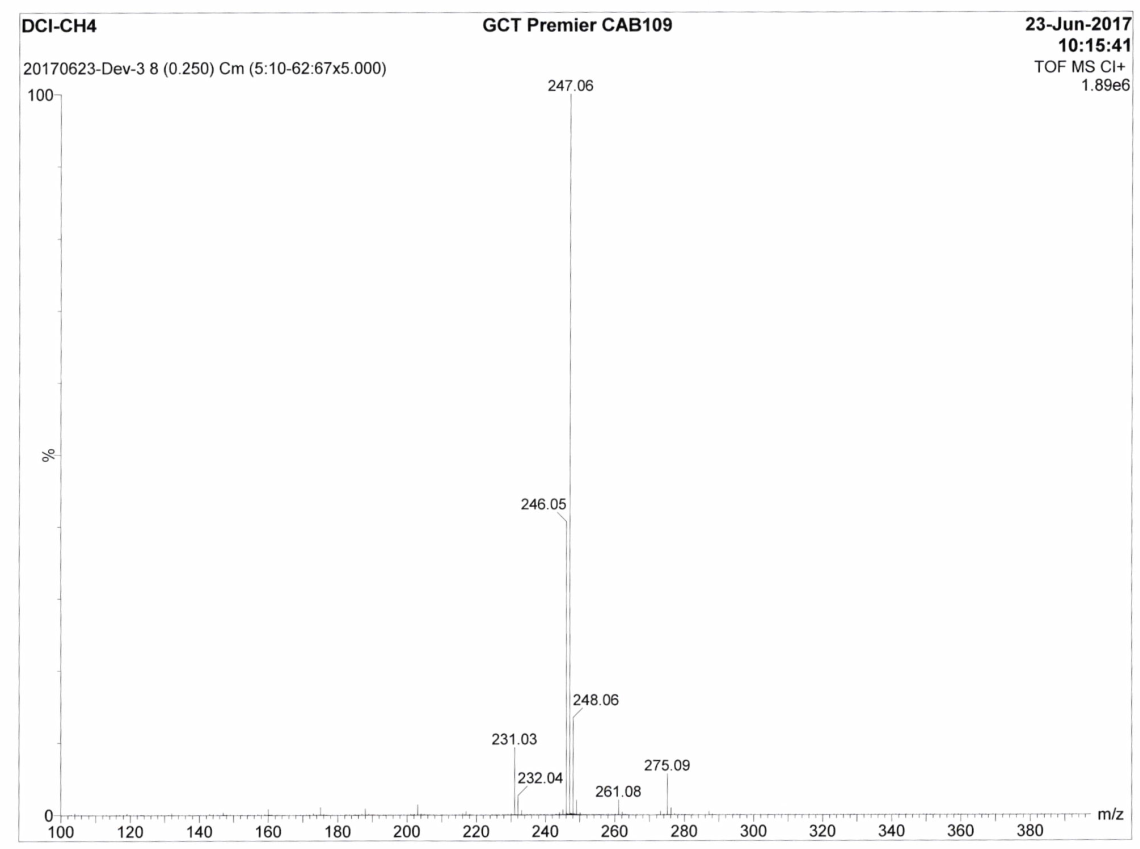


**Fig 24S. ^1^H NMR spectrum of (5) in CDCl_3_**

**
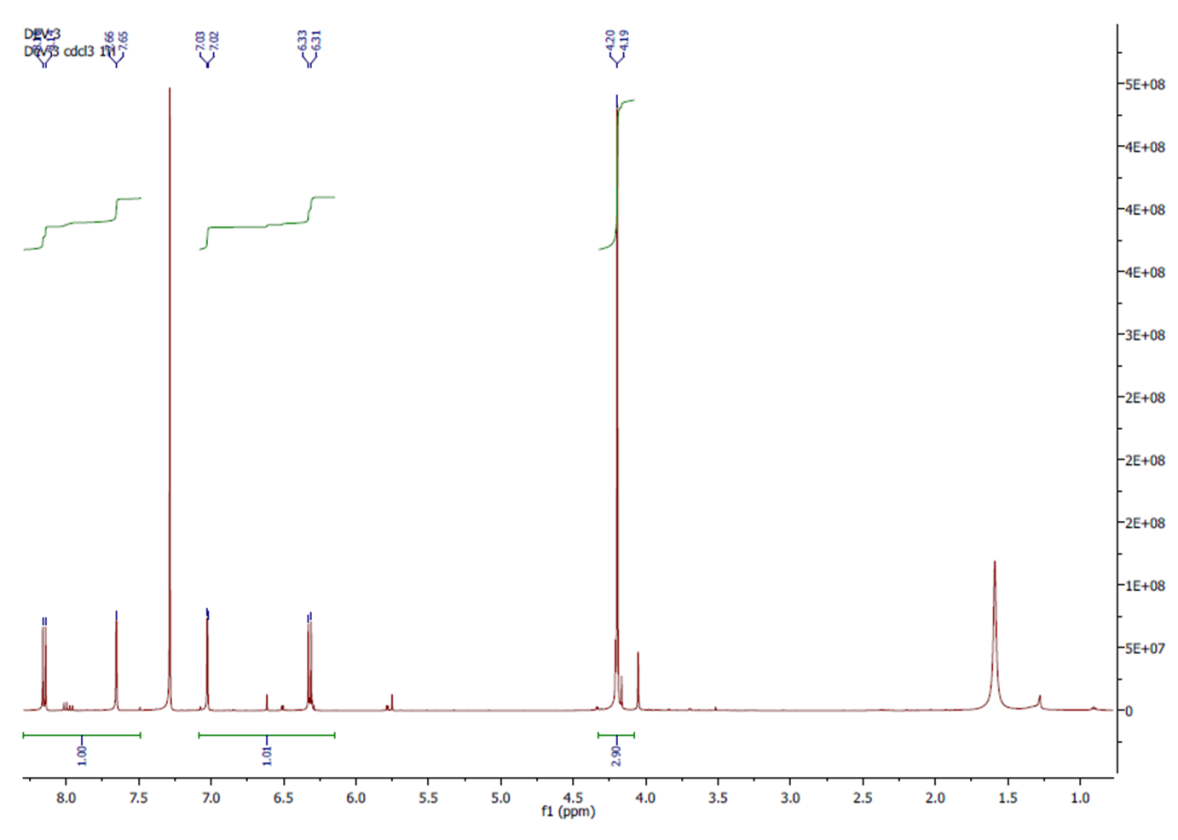
**

**Fig 25S. ^13^C NMR spectrum of (5) in CDCl_3_**


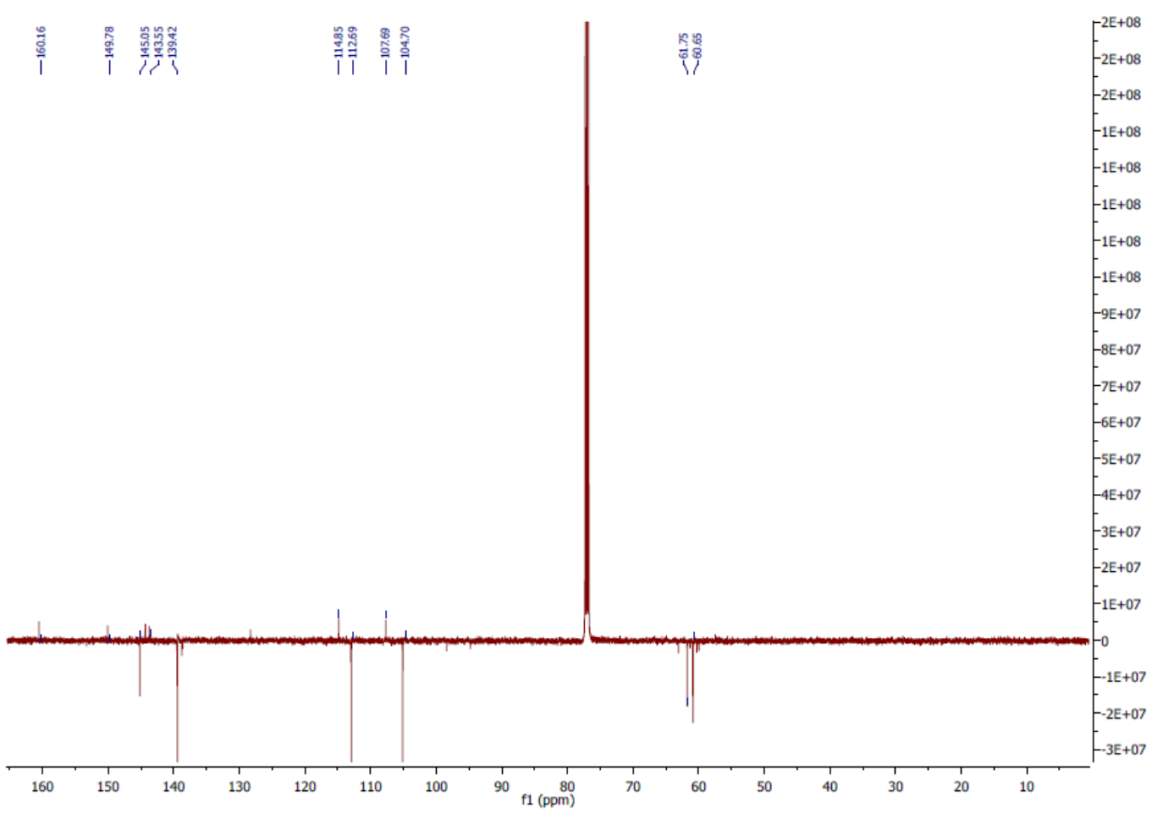


**Fig 26S. IR spectrum of (5)**


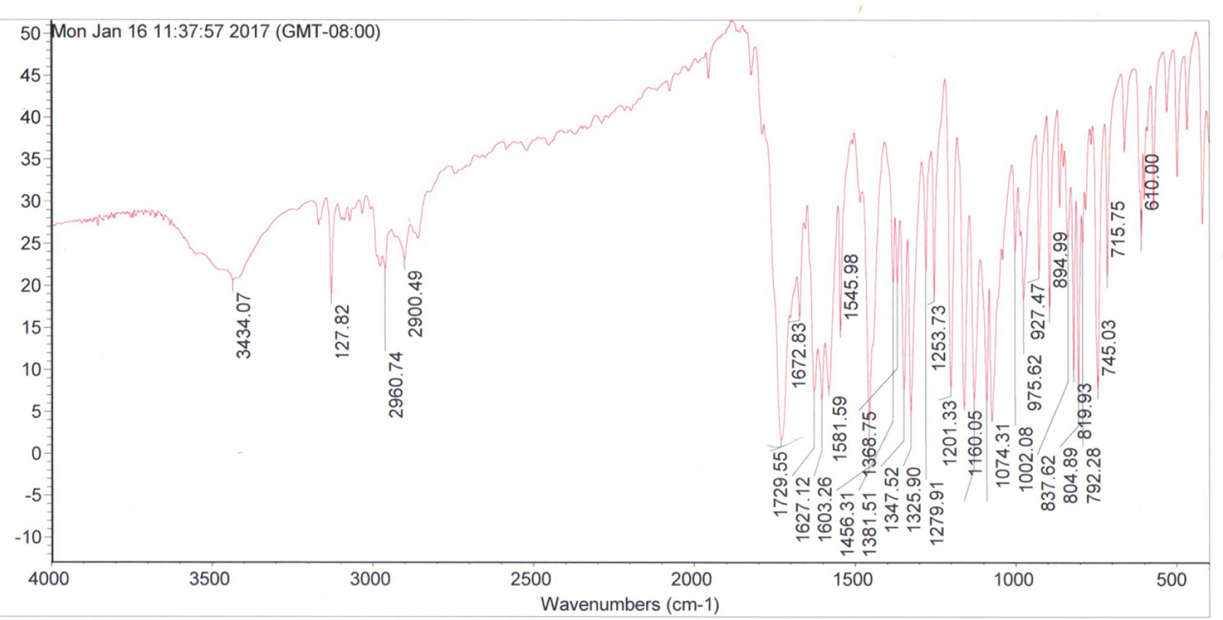

Supplement: Supplemantary_material [file mmc1.doc]
